# Learning Designers as Expert Evaluators of Usability: Understanding Their Potential Contribution to Improving the Universality of Interface Design for Health Resources

**DOI:** 10.3390/ijerph20054608

**Published:** 2023-03-05

**Authors:** Amanda Adams, Lauren Miller-Lewis, Jennifer Tieman

**Affiliations:** 1 Research Centre for Palliative Care, Death and Dying, College of Nursing and Health Sciences, Flinders University, Bedford Park, Adelaide, SA 5042, Australia; 2 School of Health, Medical and Applied Sciences, CQUniversity Australia, Wayville, Adelaide, SA 5034, Australia

**Keywords:** palliative care, usability evaluation, expert-based evaluation, expert peer review, interface design, user-centred design, Learning Designers, multidisciplinary teams

## Abstract

User-based evaluation by end users is an essential step in designing useful interfaces. Inspection methods can offer an alternate approach when end-user recruitment is problematic. A Learning Designers’ usability scholarship could offer usability evaluation expertise adjunct to multidisciplinary teams in academic settings. The feasibility of Learning Designers as ‘expert evaluators’ is assessed within this study. Two groups, healthcare professionals and Learning Designers, applied a hybrid evaluation method to generate usability feedback from a palliative care toolkit prototype. Expert data were compared to end-user errors detected from usability testing. Interface errors were categorised, meta-aggregated and severity calculated. The analysis found that reviewers detected *N* = 333 errors, with *N* = 167 uniquely occurring within the interface. Learning Designers identified errors at greater frequencies (60.66% total interface errors, mean (M) = 28.86 per expert) than other evaluator groups (healthcare professionals 23.12%, M = 19.25 and end users 16.22%, M = 9.0). Patterns in severity and error types were also observed between reviewer groups. The findings suggest that Learning Designers are skilled in detecting interface errors, which benefits developers assessing usability when access to end users is limited. Whilst not offering rich narrative feedback generated by user-based evaluations, Learning Designers complement healthcare professionals’ content-specific knowledge as a ‘composite expert reviewer’ with the ability to generate meaningful feedback to shape digital health interfaces.

## 1. Introduction

For developers of online health resources, some inherent difficulties are identifying and recruiting representatives of the intended audience to participate in usability evaluations [1], compared to products for general or commercial public consumption. For information resources serving complex health-subject domains with complicating factors, including multidisciplinary team interventions [2], patients with multimorbidity or sensitive care areas [3], recruitment becomes increasingly exigent. Strategies are also required to overcome ethical, privacy, and communication barriers to accessing, identifying, and recruiting patients or carers within healthcare settings, services, and systems [4].

Palliative care is one such domain. Palliative care is provided to patients who have been diagnosed with a non-curable life-limiting condition, illness, or disease, as a family-centred care model supporting the quality of life of the dying person until death, and provides help for carers and families during the illness and bereavement [5,6]. Therefore, the need for online palliative care information transcends cultural and social boundaries across the socioeconomic divide, is not limited to gender or age, and can support geographically isolated communities where health services are limited. This diversity in user characteristics, backgrounds, and experience demands the application of user-based usability evaluation methods during the development period to generate feedback to modify the interface to ensure that all users can find and understand the information provided to assist with decision-making for loved ones at the end of their lives.

For developers applying a user-centred approach, accessing patients receiving palliative care or their carers could influence the likelihood of usability being evaluated. Involving patients and carers in the process is challenging on three fronts: (1) identifying potential volunteers from within the community [7], specialist palliative care services or from acute care settings [8]; (2) gatekeeping protecting participation due to their perceived ‘vulnerability’ by healthcare professionals [9,10,11]; and (3) availability to be involved due to time constraints [12] or caring commitments [7].

Demographically, carers are likely to be engaged with the online environment [13] and representative of a heterogenous participant group. Carers have diverse backgrounds, ethnicities, previous experiences, digital competency, and health literacy. User interface design for digital palliative care resources is required to support end users with wide-ranging literacy levels, knowledge, information needs, and technical abilities. Consequently, involvement in evaluations can support development teams in understanding the relationship between elements of their interface design and end-user interactions to optimise the end-user experience for all users regardless of their needs, abilities, or requirements.

Where representative carers or family members as end users may not be available to participate in evaluations [14], alternative methods could be deployed as a proxy for these user-based evaluation activities [15]. Access to available experts and the selection of appropriate expert-based usability evaluation methodologies (UEMs) depends on the setting, team structure and experience (or maturity) in developing online health information resources. For commercial entities with mature development structures, teams are highly experienced in user research, with usability experts having implicit product knowledge. Subject-matter experts are adjunct to the design process and offer insights into the intended audience’s lived experiences.

For digital health resources developed from research activities, multidisciplinary development teams within academic settings are likely to be non-specialists in designing and developing online or digital health products. Subject-matter experts and academic development teams will likely adopt a collaborative approach to supplement their deficiencies in the product development cycle through contracted partnerships with individuals offering expertise in areas including technical development, design, evaluation, or marketing and promotion. As content specialists, healthcare professionals and clinicians are vested in translating their research outcomes into a meaningful experience for their patients, carers and family members. However, the involvement of usability experts is unlikely due to a lack of funds to support this expertise, along with the recognition of the need to engage in formative assessment to optimise interface design for end users.

In higher education institutions, Learning Designers consult with teaching academics to guide development, incorporate learning pedagogy, and construct measures or instruments assessing the effectiveness of materials to scaffold learners’ knowledge [16]. In an evaluation setting, understanding features contributing to end-user acceptance and the functionality of the interface design enhances the competency to identify issues or errors that contribute to interface usability levels [17]. Learning Designers, therefore, could be a source of usability evaluation expertise available to academic multidisciplinary research teams in academic settings whose projects are underfunded or under pressure, and who are inexperienced in designing and developing digital health resources for patients, carers, and families.

### 1.1. Expert and Composite Usability Methods as Alternatives to User-Based Evaluations

In contexts where representative end users may not be available to participate in evaluations [14], alternative UEMs can be deployed as a proxy for user-based activities [15]. These inspection methods involve experts applying theoretical knowledge to explore the interface from the perspective of a surrogate end user [18]. Expert feedback generated is explored to inform the reiteration of the user interface instead of usability data generated from contextualised to real lived experiences or experiential learnings of the representative end user. For multidisciplinary teams, usability evaluations of user interfaces are most likely to be conducted as expert peer reviews rather than cognitive walkthroughs or heuristic evaluations. Access to double experts, evaluators with both usability and medical or health domain knowledge is limited, and upskilling existing healthcare professionals to be competent usability experts is impossible due to time constraints or limited interest.

As usability novices [19], healthcare professionals are typically involved in a hybrid inspection approach to generate feedback on the interface by undertaking content peer review (to ensure reliability, accuracy, and quality of information) [20], whilst providing additional narratives on perceived usability errors as a surrogate end user. Considerations of interface design elements, including information structure, visual aesthetics, functionality (navigation and interactivity) and understandability of content, are assessed from the healthcare professionals’ real-world experiences of the needs and requirements of their patients, carers, or families [21]. Whether healthcare professionals with a humanistic perspective to understand patients’ or carers’ interactions based on practise experience [20] are adequate to identify most usability problems impacting end users is pertinent.

### 1.2. Heuristic Evaluations, Cognitive Walkthroughs, Compared to Expert Review

Heuristic evaluation and cognitive walkthroughs are other inspection UEMs where experts detect potential usability issues during sessions. Heuristic evaluations require experts to assess the interface’s usability against a set of guidelines or principles [22,23]. Cognitive walkthroughs invite experts to undertake activities within the interface, to behave like a user relative to their cognitive model of the resource, informed by objectives or needs and knowledge [22,24]. Research indicates that development teams may need to consider whether end-user experience levels should influence the choice between heuristic evaluations and cognitive walkthroughs [25].

Expert peer review also considers end users’ knowledge and awareness of the subject domain and specific content-based errors detected by domain specialists based on the understanding and context of the objective and application of information provided within the resource [26]. All three methodologies require experts to have subject domain knowledge and an awareness of how end users will behave within the interface. Only the heuristic evaluation requires participant evaluators to be double experts who are either experienced or trained by resource developers in assessing usability and are specialists in content [22,27].

### 1.3. Expert UEM versus Gold Standard ‘Usability Testing’ Method

For all development teams, usability testing is considered the ‘gold standard’ [28], a method crucial to strategically reiterate the interface centred on the needs and technical abilities of the intended audience [29]. Usability testing is an empirical UEM assessing the efficiency, effectiveness, and satisfaction of interface interactions as a reflection of cognitive processes driving end-user interactive behaviour [30]. Feedback to inform reiteration of the user interface is generated from the end user’s ability to identify usability errors relating to an individual user’s characteristics, context, and use environment [30].

Pragmatically, the likelihood of multidisciplinary academic development teams conducting usability testing depends on funding, time available, or the availability or experience of project staff to recruit, conduct, interpret and report outcomes within constrained processes. Moreover, unlike commercial developers with a specific audience, the generalist nature of health resources forces additional complications upon developers to discern range and prioritise selection, accounting for a diverse audience’s knowledge, skills, and abilities.

In comparison, expert-based usability inspection methods can offer a high rate of return for a relatively small investment of time and money [25,31] in comparison [30] whilst outsourcing usability expertise to specialists. Expert inspectors identify between 30–60% of errors [32]; on average, 49% of common errors are shared between methodologies. However, double experts do not have the ability to emulate errors associated with critical end-user behaviours [20], resulting in a high frequency of false positives identified, non-veritable errors for end users and missing errors, severely impacting end-user interactions with the resource [32,33].

### 1.4. Learning Designers as Double Composite Experts for Usability Evaluations

The difficulties experienced by multidisciplinary development teams underpinned by usability evaluation inexperience and practical constraints surrounding development processes could be offset by the involvement of an ‘in-house’ composite double expert. For example, accessing Learning Designers already employed within higher education settings could offer development teams the ability to examine interface flaws objectively, comparing content to domain knowledge to judge quality, reliability, and accuracy. Further offering feedback as a subjective assessment of the interface for technical errors associated with operational or functional aspects that decrease usability and impede end-user interactions. How this feedback compares to the quality or efficacy of end-user-derived usability feedback is unknown.

As the literature has not previously reported recruitment, participation, or outcomes from usability evaluations of digital health information resources undertaken by Learning Designers during development, this current study will explore the following research questions:

RSQ1. How does usability feedback differ between Learning Designers and healthcare professionals when completing an expert review of a palliative care resource?

RSQ2. Is there a role in usability evaluations of digital health interfaces for Learning Designers: as a sole expert evaluator group, in combination with healthcare professionals, or a tripate with healthcare professionals and end users?

## 2. Materials and Methods

### 2.1. Study Description

Traditional usability practise requires developers to recruit three to five heuristic double experts [34] with domain content knowledge and experience or training in usability. Given the scarcity of experts in the health domain with usability expertise, recruiting representatives from both reviewer groups could balance the identification of both content-based and usability errors whilst countering any perceived weaknesses in each group’s ability to detect interface issues, especially in scenarios where end users are deficient in the process. Therefore, the primary objective of this study is to investigate the feasibility of involving subject-matter experts (healthcare professionals) and digital experts (Learning Designers) to explore their availability for recruitment and the appropriateness of their feedback to reiterate a palliative care resource interface through error identification. A novel hybrid approach to evaluation was utilised, combining expert peer review and the cognitive walkthrough evaluation process to generate feedback from the expert evaluators.

### 2.2. Prototype Development and Overall Evaluation Approach

Comprehensive usability evaluation was undertaken on an early prototype of the Australian Carer Toolkit for Advanced Disease (known here as the CarerHelp Toolkit or ‘the Toolkit’). The Australian Government Department of Health funded the Toolkit project. The CarerHelp Toolkit is an online resource designed to support family carers of relatives or friends with the advanced disease living at home within the community. This resource aims to increase family carers’ knowledge and confidence to support the provision of end-of-life caregiving at home through evidence-based information, educational activities to build skills or knowledge, and access to how-to guides in the form of vignettes, interactive activities, and videos.

Usability evaluation approaches from the Web Development Model for Healthcare Consumers (WDHMC) [20] were applied to the prototype. This tripate included user-based (usability testing), expert-based (expert peer review) and content-based evaluation methodologies deployed during the development phase. With the current study focusing on outcomes from expert peer review and errors identified by carers within the usability testing process, the primary objective is to explore the feasibility of involving Learning Designers in usability evaluations of digital health resources.

### 2.3. The Palliative Care Website Prototype

**Figure 1:**
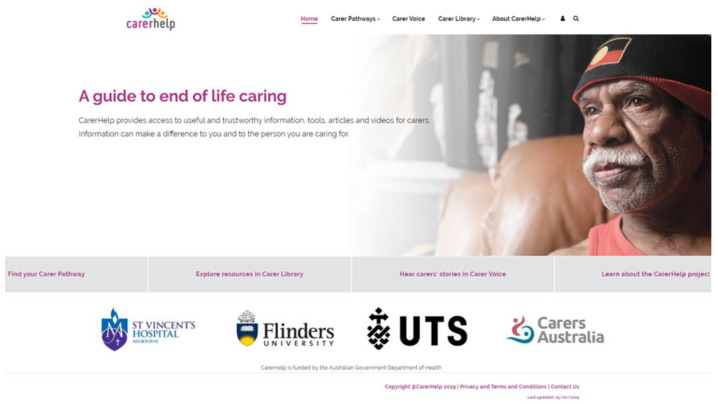
The Homepage of the Carers Toolkit prototype.

**Figure 2:**
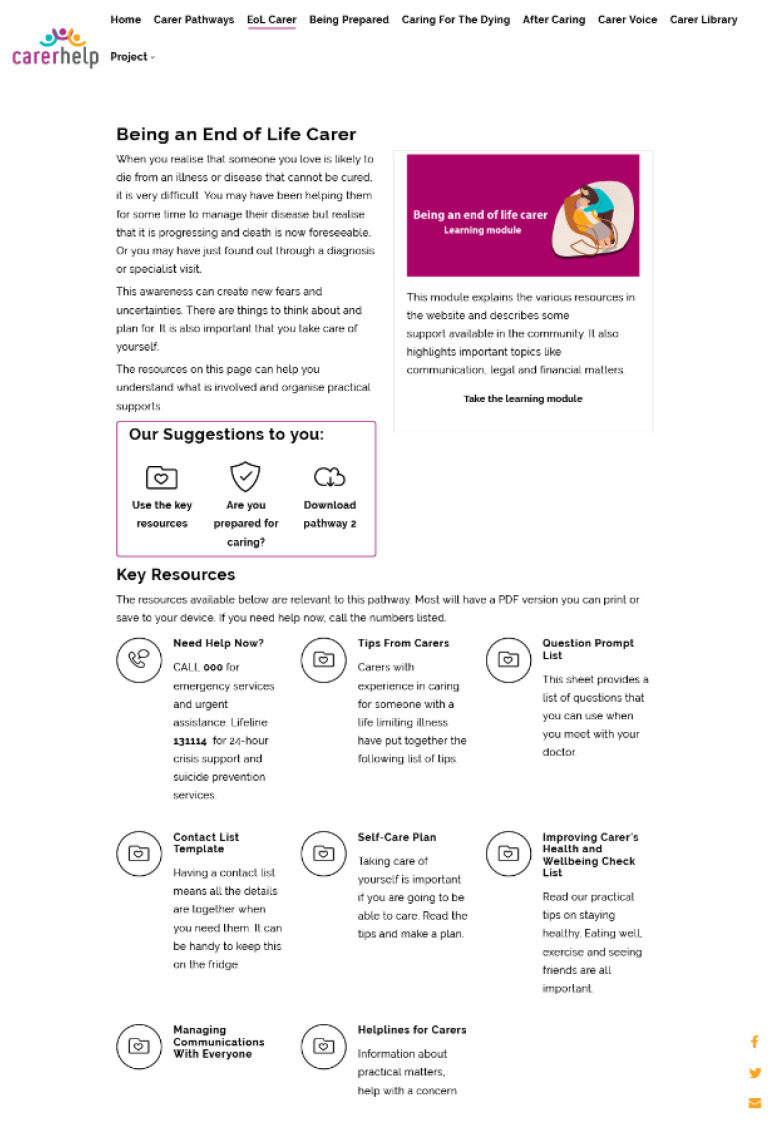
An example of the internal content page of the Carers Toolkit prototype.

Usability evaluations were conducted on a prototype of the CarerHelp Toolkit; information was structured across two levels consisting of four sections: Carer Pathways, Carer Voice, Carer Library and About CarerHelp. User-based usability testing was undertaken on an earlier version of the prototype accessed by experts due to the timing of evaluation within the development cycle. These prototypes were similar, although the earlier version had fewer interactive features and limited visual representations within the interface compared to the later version. Screengrabs are provided in Figure 1 (Homepage) and Figure 2 (internal webpage of the prototype version) of the Toolkit prototype as evaluated by the expert evaluator groups.

The reiterated post-release version of the CarerHelp Toolkit is freely available online at the URL (accessed on 10 January 2023): https://www.carerhelp.com.au/.

This study received ethical approval from the Flinders University Social and Behavioural Research Committee (Project Number 8347).

### 2.4. Expert Review Methodology

Two groups of experts, healthcare professionals (HCP) working in palliative care as subject-matter experts and Learning Designers (LD) as practising designers of digital education (having interface design, usability awareness and interaction experience—described as ‘digital expertise’), were invited to participate as reviewers within this study. Suitability for the study was limited to professionals currently practising and there were no limitations placed on levels or years of experience. Instead, inclusion criteria focused on availability to be involved around regular working hours, access to a device with Internet connectivity, and the willingness to share feedback in written and verbal formats (using online conferencing software). Between 4 and 8 reviewers were sought for each review group to ensure reviewer diversity (areas of expertise and individual characteristics). As this was an exploratory study, a smaller sample for each group was considered acceptable, especially given the difficulties in identifying and recruiting representatives from both professions (palliative care clinicians and Learning Designers). Expert reviewers could not participate in the study due to a lack of flexibility in their work schedules rather than disinterest.

#### 2.4.1. Evaluator Group—Palliative Care Healthcare Professionals

Local, state and national palliative care organisations/services were approached to identify potential HCPs who had previous experience or were currently supporting the palliative care needs of patients and their carers living within a community setting. Palliative care specialists, general practitioners, nurses, and allied health professionals were invited to participate in the usability sessions through their services. These included Southern Adelaide Palliative Service, Palliative Care Queensland and the Australian and New Zealand Society of Palliative Medicine. Four HCPs who were actively involved in palliative care practise consented to review the Toolkit. Participants included two directors of palliative care services (also providing care as a general practitioner and nurse), a social worker and nurse practitioner.

#### 2.4.2. Evaluator Group—Digital Experts: Learning Designers (LDs)

The professional organisation for online designers (learning, educational and instructional) working within the private and higher education sector, the Australasian Society for Computers in Learning in Tertiary Education (ASCILTE), assisted by widely promoting the study to their membership. Seven LDs from across Australian higher education institutions self-nominated and consented to be an expert reviewer.

### 2.5. Expert Review Protocol

Before commencing the review process, participants provided informed consent after the researcher had explained the research protocol and perceived risks involved. Each expert was then asked for descriptions of their professional credentials (professional title, professional practise setting and post-qualification years of experience (working as HCP or LD) and to self-assess their level of technical ability using the Internet by responding to the following question:

I am:

- An avoider of everything online
- A novice or learner or beginner
- Mostly confident—having intermediate skills
- An expert who is confident in finding and using online information

Evaluators completed the review process in two stages; the first was a digital document providing a structure and guiding interaction with the prototype (refer to Supplementary File S1). Professionals were asked to comment and record their thoughts on content, navigation, interface features, interactive activities, or widgets, including what they determined necessary for the end user. All expert reviewers were invited to provide (as much or as little) feedback as they liked and were not limited to the guiding questions or statements within the feedback document. Although some activities embedded within the Toolkit were out of the scope of the review.

Once the review document was completed and returned to the researcher, participants undertook the second stage of the review process by remotely debriefing their findings during a 30 min online interview session and providing an opportunity to explain their written feedback. In addition, the functionality of the conference software [35] demonstrated visual issues critical to function, incorrect or non-sensical in the context of the content, information flow and navigation across and within pages of the Toolkit.

### 2.6. User-Based Evaluation Methodology

User-based feedback on issues and errors within the prototype was generated through formal usability testing methodology, with participants representing the intended audience of the CarerHelp Toolkit.

#### Usability Testing Participants

Family or primary carers of patients with palliative care needs living at home within the community were invited to participate in the study. Carers were sought from the wider community and specialist palliative care services within the southern areas of Adelaide, South Australia. A cohort of active and bereaved carers (6–8 months post-death) was identified by the Network Facilitator at the Laurel Hospice Caregiver Network (Southern Palliative Care Service, Adelaide). The researcher contacted carers who were interested and eligible to participate, and the study protocol was explained. Perceived risks were clarified, and formal consent to participate in usability testing was provided. A sample size of six was calculated by applying the probabilistic model of problem discovery [36] to identify interface errors occurring 50% of the time at a level of error discovery of 98% [37,38].

All participants customised the device and peripherals, reflecting their natural Internet interaction environment experienced at home (for example, using desktop or mobile, mouse or trackpad and screen augmentation). All online interactions were documented and digitally recorded using conference software to ensure that all audio commentary, facial expressions, and accompanying cursor movements were captured for post-session analysis.

### 2.7. Usability Testing Session Protocol

The following section will not comprehensively describe the usability testing protocol or methodology. Instead, detailed explanations of scenario development, task descriptors, interface satisfaction assessment, and outcome performance measures are described in the formal usability report informing reiteration of the interface (refer to Supplementary File S2).

Before beginning the session, the researcher ensured that each carer was aware of the Toolkit content and any perceived risks and emphasised that the session could be stopped at any time if individuals were experiencing distress. After providing formal consent, carers completed questionnaires describing their online behaviours, self-rating their technical ability and level of health literacy.

Each of the six participants then completed eight scenario-based tasks within the CarerHelp Toolkit interface using the Concurrent Think Aloud technique, where individuals verbally describe their thoughts or feelings when completing each task. Participants completed an identical set of tasks during the sessions; each task had a completion limit of 3 min. Completion was when the target information was located within the allowed time. Failures were registered if the target was not found within time, if the incorrect target was identified or if the participant abandoned the task.

### 2.8. Data Analysis

Methods undertaken within this study follow traditional formative usability evaluation processes requiring small groups of evaluators to provide reiterative feedback to improve interface design. Therefore, it is acknowledged that this study is under-powered and unlikely to detect statistically significant differences between evaluator groups due to the small sample sizes. However, trends in descriptive usability error detection patterns and exploration of types or severity of usability errors can assist in understanding the potential roles LDs can play in optimising digital interface designs for palliative care resources (and health interfaces more widely).

#### 2.8.1. Expert Evaluator Feedback

After deidentifying data, a qualitative meta-summary of content findings was generated from the written feedback document and other narratives from the debrief interviews from both reviewer groups. Quantitative logic was then applied to aggregate error types between participants, and this provided a process to assess error frequency and identify problems, missing resources or content, and opportunities or suggestions for interface improvements. Types of errors describe problems experienced by evaluators relating to specific functional aspects of user interaction at the user interface level.

Further analysis of content-specific errors identified within written information included frequency-based analysis of the types of content errors detected by reviewers by applying a modified coding schema to accommodate the interface’s online environment and technological aspects to error data as described in Table 1 (as compared to Sayoran’s original schema [39] for revision of written text).

#### 2.8.2. Meta-Aggregation of Usability Errors across Review Groups

All types of errors from all three reviewer groups (USER (carers), HCP and LD) were collated into a single list, with a bottom-up approach to meta-aggregation adopted to collate the differences from summarising types of usability errors detected within the Toolkit interface across all evaluators.

Aggregation provided an opportunity to compare commonalities or differences in error types identified by specific reviewers, further providing the capacity to highlight interface problems discovered exclusively by a single reviewer group or perceived (shared) across more than one reviewer group. Error types were grouped into classes categorised by approaches, development considerations, content level or structural elements of the user interface. These are considered groups of errors relating to global aspects of user interface design.

Classes of usability error included:

- Content-specific
- Design or content construction
- Information flow
- Navigation
- Embedded resource or activity
- Pedagogy or educational strategies
- Minor typographical or grammatical errors
- Major typographical or grammatical issues

The severity of interface errors is then assessed on three factors [40] influencing usability:

- Frequency of the occurrence within the interface
- Impact of the error (if it occurs) on users to overcome
- The persistence of the error within the interface continuously affects evaluator interactions

In considering these factors, each error was assessed and, using Nielsen’s Severity Rating scale [40], received one of the following severity ratings:

0 = I do not agree that this is a usability problem at all

1 = Cosmetic problem only: need not be fixed unless extra time is available on the project

2 = Minor usability problem: fixing this should be given low priority

3 = Major usability problem: important to fix, so should be given high priority

4 = Usability catastrophe: imperative to fix this before a product can be released

In rating interface issues, errors persistently having a significant impact on evaluators received the highest severity rating.

## 3. Results

### 3.1. Demographics of Expert Reviewers

#### 3.1.1. HCP Demographics

Of the four HCPs recruited for the study, two were from Palliative Care Queensland and one from the Australian and New Zealand Society of Palliative Medicine and Southern Adelaide Palliative Care Service. The HCPs had a minimum of seven years specialising in palliative care (range 7–20 years, median = 12 years). Three of the four self-rated themselves as having expert technical skills, with one self-rated their ability as being of intermediate level. Characteristics of the HCPs are presented in Table 2.

#### 3.1.2. LD Demographics

Seven Learning Designers were recruited in total, all members of the ASCILTE organisation. All participants were employed within the university sector and held positions in institutions within five different states of Australia. All LDs were self-assessed as experts in using technology whose combined experience spanned 24 years post-qualification (range 3–27 years, median 12 years). In addition, two participants were managing academic units; although they had extensive experience as educational technologists, the other five LDs actively practised the design of educational materials. Characteristics of participants working in higher education as professional designers of digital learning experiences working are summarised in Table 3.

### 3.2. RSQ1: Comparative Analysis of Usability Error Data from Expert Evaluators

The differences between feedback generated through expert review UEM by subject-matter (HCP) and LD with digital expertise were examined. The types, frequency and severity of usability errors detected by each evaluator group were examined to explore areas of strengths or weaknesses when detecting problems within the interface. Additionally, content-specific and types of qualitative descriptions of errors were compared between groups to supplement the analysis of usability problems detected by each set of evaluators.

#### 3.2.1. Analysis of Frequency and Usability Error Types Detected by Expert Reviewers (LD versus HCP)

In total, both expert evaluator review groups identified 279 errors within the Toolkit prototype interface (all data are presented in Table A1—Appendix A). LDs found 202 (72.40%) errors, with each designer detecting a mean (M) = 28.26 errors, compared with HCPs, who identified 77 (27.60%) errors at an average of 19.25 errors per reviewer. It was acknowledged that due to the small numbers of participants, there was difficulty in ascertaining existing statistically significant differences between error identification and years of experience for each evaluator. However, there were trends in the types of errors identified, frequency and severity of errors which can be observed within the data collected.

For HCPs, years of experience did not reflect an increase in the number of errors detected; however, data suggested an inverse trend where newer HCPs were more adept at identifying more errors within the interface (MHCP 6–10 years = 22 errors, 57.1% total: MHCP 11–15 years = 1 error, 22.1%: MHCP 16–20 years = 16 errors, 20.8%). Those HCPs who had been practising palliative care for 6–10 years identified the highest number of errors with a medium–low severity compared with other cohorts. In addition, 79.2% of interface errors were detected by HCPs who were self-rated experts with technology compared to intermediate-skilled HCP reviewers (20.8%).

For LDs, years of experience designing online positively influenced error detection frequency. Although LDs with greater than 16 years of experience identified an equivalent number of errors as those with less experience (104 errors, 51.49% versus 98 errors, 48.51%), LDs with increased practical experience on average detected 52 errors compared with M = 19.6 errors for LD with less than 16 years designing experience. LDs also identified a higher frequency of errors rated highly severe than errors found by HCPs, with 71.4% of the most severe errors detected by LDs with between 11–20 years of experience. Errors with the highest frequency were proportional to LD and HCP reviewer groups when calculated as a percentage of total errors identified. Specific content errors constituted over 50% of total errors detected by HCP and 38.1% for LD. The frequency of navigation issues was comparable between 14–15% of total errors per group, and LD identified a greater proportion of errors impacting information flow (18.3% total errors) than the HCP reviewer group (11.7% total errors).

#### 3.2.2. Comparison of Content-Specific Errors Identified by HCP and LD Reviewers

**Figure 3:**
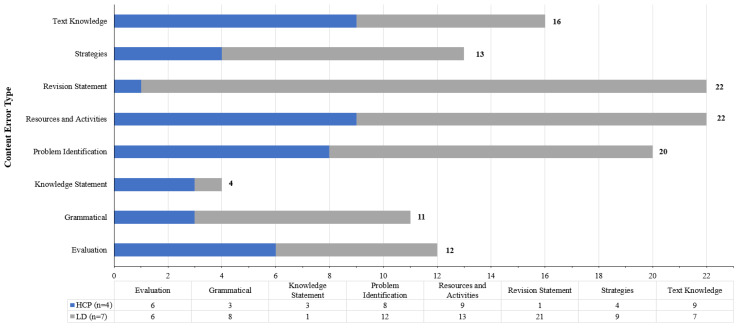
Specific content errors identified by healthcare professionals and Learning Designer evaluator groups.

Analysis of the qualitative feedback was undertaken by the primary researcher (AA) and categorised the content based on the modified Sayoran’s schema provided in Table 1. Categorisation of the feedback provided by the eleven expert reviewers identified a total of 120 content specific errors categorised into eight error types. Results are displayed in Figure 3. A detailed summary of feedback types and example descriptions are presented in Table A2—Appendix A).

Whilst LDs detected a greater frequency of errors than HCPs overall (77 errors, 64.2% versus 43 errors, 35.8% respectively), average errors per reviewer were similar across groups (MLD = 11.0, SD = 5.9 versus MHCP = 10.8, SD = 3.1). Types of content errors with the greatest frequency within the interface by experts explicitly referenced issues within embedded resources or learning activities (18.3%, (5) in Table A2), for example:

*“Need to make sure that this toolkit provides information for carers on how to improve and sustain person’s quality of life when at home”* (HCP reviewer),

descriptions of errors requiring rephrasing with examples of revisions statements (18.3%) such as:

*‘Caring for someone who is dying is the end of a journey of caring …—could be something like: Caring for someone dying also means that your role of carer will come to an end after the person has died. These resources help you to be prepared for dealing with the end-of-life care’* (LD Reviewer),

an explicit reference to problems within the interface (16.7%), including:

*‘I think language is okay, but there are just too many words’* (HCP Reviewer),

Or:

*‘…appropriate to also insert a link here to take the users back to the first page, rather than telling them to go to and use the menu (where is that?) to get back to the main page.’* (LD Reviewer).

HCPs and LDs detected equivalent numbers of error descriptions with a provision of evaluation statements and assertions on the learnings from the text. LDs identified a greater frequency of errors describing grammatical or spelling errors (3 HCP:8 LD), were more likely to provide suggestions on applying strategies to content (4 HCP:9 LD) and were more forthcoming with the provision of alternate text through revision statements compared with healthcare professionals (1 HCP:21 LD). Unsurprisingly, HCPs identified specific issues or errors within the written content of the Toolkit webpages and were skilled at detecting content mistakes in-text to provide feedback based on statements of their knowledge of palliative care.

### 3.3. RSQ2: The Acuity of Expert Usability Error Data Was Compared to End-User Usability Error Data Generated from Formal Testing of the Prototype Undertaken with Primary Carers as Representatives from the End-User Group

Analysis examined the frequency and types of errors identified by each evaluator group. The role of LDs as evaluators in designing digital palliative care resources was explored through comparisons of LD usability data across evaluator groups, both as an independent reviewer group and in combination with the other evaluators. Shared (mutually inclusive) and exclusive errors were mapped to understand the influence that different evaluators have on usability data collected as a pragmatic reflection of the availability of these groups to participate in evaluations during the development process undertaken within academic development settings.

#### Evaluator Group Comparisons between LDs, HCPs, and USERs (Carers)

Similarities and differences in the error type and frequency of detection by reviewer groups involved within the Toolkit usability evaluation approaches were explored by meta-aggregation of HCP, LD, and USER data to characterise error occurrences within the interface. Errors were categorised by reviewer, type, level of exclusivity or inclusivity and whether the errors are unique or co-existing across the reviewer group. Unique errors are discernible occurrences identified by single or multiple reviewers whose accretion decreases total interface error counts compared to the overall error number.

**Figure 4:**
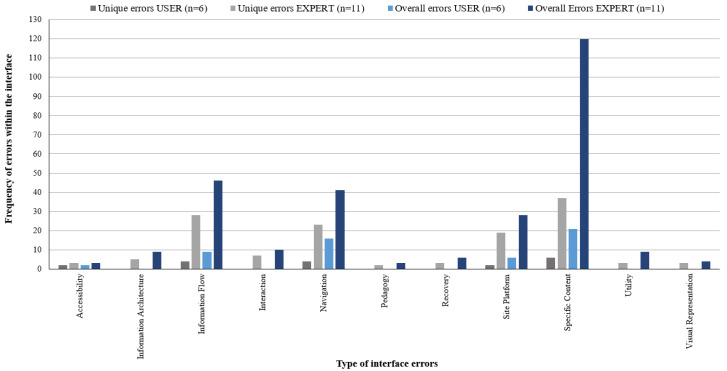
Frequency of overall and unique errors identified by carers (USER) and EXPERT (HCP and LD) evaluator groups.

Seventeen reviewers identified a total of 333 errors that did not occur exclusively for any single reviewer group, and these are co-existing within the interface and identified by all three reviewer cohorts. Further analysis found 167 unique errors occurring once within the interface (error data summarised in Figure 4, data presented in Table A3—Appendix A).

The HCP reviewer group identified similar frequencies of co-existing and uniquely occurring errors (23.12% and 25.15%, respectively), whilst the USER group detected 10.78% of unique errors, although only 16.22% of the overall errors within the interface. LDs identified the greatest number of errors overall and unique errors at the highest frequency within the interface (202, 60.66% and 107, 64.07%, respectively) compared to the other reviewer groups. LDs also detected higher frequency on average per reviewer in both inclusive (MLD = 28.86: MHCPs = 19.25: MUSER = 9.0 errors) and uniquely occurring issues (MLD = 15.29: MHCP = 10.0: MUSER = 3.0 errors). Differences in the rate of error identification between the USER and expert reviewers widened when combing error counts into a single EXPERT cohort (HCP + LD). The EXPERT group attributed 279 errors (83.78% of the total) and 133 uniquely occurring errors (88.10% total) at an average of 12.09 errors per expert.

**Figure 5:**
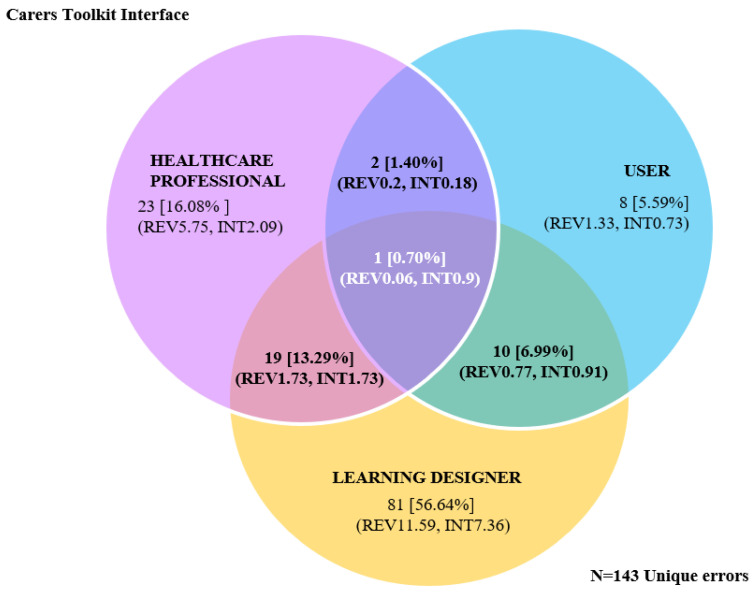
Unique exclusive and mutually exclusive interface errors identified between and across evaluator groups. Number of unique errors (%Total) (REV = average unique errors identified per reviewer, INT = average unique errors identified by reviewer group across the interface).

**Figure 6:**
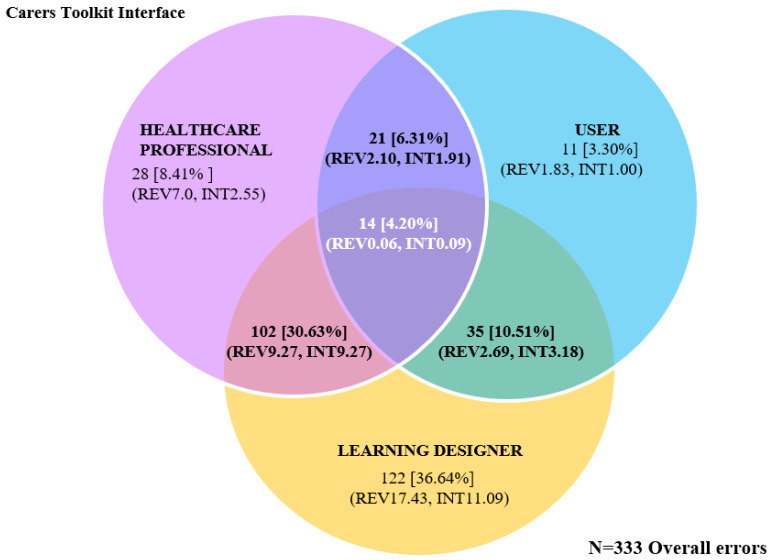
Overall errors interface errors identified between and across evaluator groups. Number of overall errors (%Total) (REV = average errors identified per reviewer, IN = average errors identified by reviewer group across the interface).

Exclusive and mutually inclusive errors were identified across and between reviewer groups (data are presented in Table A4—Appendix A), and the total overall error count remained constant (*N* = 333). Further data consolidation into combinations of reviewer groups where each error instance is assigned a single identifying group decreased the overall unique error count from *N* = 167 to *N* = 143. Similarly, the analysis found that LDs identified over 50% of the total unique errors compared with HCPs (16.08%) and 5.59% for the USER review group. Consequently, review groups in combination with LDs were more likely to identify an increased proportion of unique errors compared to other HCP or USER group combinations (uniquely exclusive and mutually errors are mapped between and across evaluator groups in Figure 5, and Figure 6 displays overall frequency of errors identified).

This pattern is analogous to counts of overall co-existing errors. LD was also more likely to identify errors or issues with site or platform performance and accessibility for end users. Five distinct accessibility issues were detected by just the LD reviewers and the LD + USER group. Differences in error detection rates between groups were analysed using independent t-tests (homogeneity of variance as assessed by Levene’s test for equality of variances and where failed, t-test for equality of means was conducted using the Welch–Satterthwaite method), significance was indicated when *p* < 0.05 at 95% confidence level. LDs identified, on average, a significantly greater number of overall errors within the interface than all other reviewer groups except for the HCP + LD review group: HCP (t(11.361) = −2.460, *p* = 0.031), USER (t(10.521) = 2.983, *p* = 0.031), HCP + USER (t(13.25) = 2.545, *p* = 0.024), LD + USER (t(20) = 2.144, *p* = 0.044) and HCP + LD + USER (t(20) = 2.747, *p* = 0.012).

LDs could also identify a significantly greater number of unique errors within the interface than all other reviewer groups: HCP (t(12.043) = −2.204, *p* = 0.048), USER (t(10.707) = 2.864, *p* = 0.016), HCP + LD (t(11.402) = 2.392, *p* = 0.035), HCP + USER (t(10.555) = 2.573, *p* = 0.01) and LD + USER (t(10.662) = 2.788, *p* = 0.018) and HCP + LD + USER (t(10.032) = 3.191, *p* = 0.01). Significant differences were also observed between the average number of overall errors detected by the HCP reviewer group compared to HCP + USER (t(10.55) = 2.573, *p* = 0.027) and HCP + LD + USER groups (t(10.309) = 2.712, *p* = 0.021). There was only a single error type that all three reviewer groups identified. An error relating to grammatical or spelling errors specific to content within the pages or activities appeared in fourteen instances across the interface and was mutually inclusive to all reviewers participating in the evaluation process.

## 4. Discussion

For digital health or medical information resource developers, there are inherent difficulties in undertaking inquiry-based usability evaluations that generate crucial expert or user-based feedback to inform interface reiterations. Factors such as identification, access, and availability of suitable experts or representative end-user recruitment influence evaluations’ likelihood of integration into typical development processes. The involvement of double experts who are equally knowledgeable of clinical subject matter and skilled in usability to evaluate digital health interfaces heuristically is appealing to development teams as it can alleviate the need for end users [41,42] to participate in the process. However, the ability to find and afford to engage these experts in development processes is limited in academic settings.

Access to LDs, a potentially rich source of technically skilled professionals with background knowledge of user-centred design and an understanding of usability evaluation approaches, would be advantageous, especially for inexperienced, under-resourced development teams. Universities engage LDs to work across interaction, visual and education design. Alignment between designing educational materials, Toolkit instructional components [43] and evaluation practise could ideally position LDs to assist in developing digital health products or resources in multidisciplinary settings. LDs understand features contributing to end-user acceptance and functionality of the interface design enhances the team’s ability to identify issues and resolve these that contribute to levels of usability, especially if end users are unavailable. Researchers are increasingly adopting usability evaluations within their typical designing approaches when creating within the digital environment, especially with the emergence of highly immersive technologies. These include virtual, augmented, or mixed reality systems and the gamification of educational resources or interactive platforms [44]. Involvement and the types of feedback generated by LDs involved in usability evaluations as experts in their own right are less clear within the literature.

In this current study, there was an opportunity to explore the potential value to multidisciplinary teams in having access to ‘composite heuristic experts’ to improve interface designs for diverse audiences by assessing a palliative care prototype. Inviting LDs to be involved in evaluations provided a unique opportunity to compare types, frequency and severity of errors identified between evaluator groups through a hybrid UEM process (modified cognitive walkthrough/expert review) to assess usability within an academic setting. The interface of the palliative care digital Toolkit was assessed by HCPs (as subject experts), LDs (interaction experts), and representatives end users (carers). The potential role in evaluation and suitability as a double heuristic expert was explored by analysing the types, frequency and severity of usability errors identified by LDs, then comparing these to those detected by subject-matter experts and users. Areas of overlap and exclusion were mapped to understand the composition of evaluators and end users required to be involved to optimally shape the interface design for a resource supporting a diverse audience. Firstly, it is important to highlight the continuing difficulties in recruiting end users and evaluators, including LDs, to participate in usability evaluations.

### 4.1. Ease of Recruitment of Experts and End Users

Difficulties in recruiting representative end users from health-specific domains were a primary driver in exploring the relative ease of identifying experts involved in the process compared to end users, for this study being carers providing palliative care within the community. Although approaches were similar, expert reviewers were equally challenging to recruit, not through lack of interest or willingness to participate but rather an availability constrained by time due to work commitments. Just as the unmoderated and remote review increased opportunities for participation in both groups of experts (as geographically distanced members were technically able to negotiate various platforms and software required for activity completion), this method could also limit participation given the diversity in user characteristics impacting interactive behaviours, especially within HCPs. Interestingly, HCPs with lower technical or digital competency levels should be encouraged to participate as a reviewer for developers to understand some of the difficulties or barriers their users could face when interacting with their interface.

Conversely, end users were extremely difficult to recruit. Promotion through carer peak body organisations electronic communication channels failed to identify any potential carers interested in participating in the study from within the local carer community. A gate-keeper advocate provided access to interested carers from the local palliative care community service. No fewer than twelve active and recently bereaved primary carers were approached from within the carer network to satisfy the usability quota of six participants. Those who did not want to be involved acknowledged that their willingness to participate was tempered by anxiety and fear of reliving painful or sad experiences rekindled by viewing the Toolkit content. These feelings are offset by the need to assist developers in improving the resource to help carers have all the missing information that could have been of value during their own experience. Active carers expressed that time and availability due to their caring duties were also reasons to consider participating in the study. The difficulties in accessing suitable participants within vulnerable populations, including from within the palliative care domain, subsequently influenced the ability to recruit without impacting the timeliness of the development process. Once recruited, both evaluators and end users were pleased to be involved in the evaluation process, offering valuable feedback on the usability of different aspects of the Toolkit prototype interface. Finally, the analysis of verbal feedback demonstrated different approaches undertaken by healthcare professionals and LDs when providing expert feedback on the errors or problems encountered within the interface.

### 4.2. The Types of Feedback Provided

Unsurprisingly, subject-matter experts (HCPs) were more likely to identify content-specific errors provided as statements of their palliative care knowledge in supporting carers’ needs in their professional practise. HCPs view the structure of sentences, text organisation and the relationship to how the text relates to a specific purpose or audience by applying process knowledge (competencies, motivations, and strategies as the reader) with metacognition to review text for issues [45] to ensure reliability, accuracy, and quality of information [20].

Learning Designers could also identify content-based errors, although their feedback offered alternatives or rephrased content as revision statements described in their review documentation. Narratives offered an insight into lay understandings of palliative care that were less detailed than the commentary offered by HCPs and should be privileged. This information potentially improves interface reiterations creating understandable content similarly levelled at an audience with a limited understanding of palliative care as part of their lived experience. Feedback provided represented a comparison of reviewers pre-existing knowledge to the overall text from a generalist viewpoint compared to the HCP process, who approached the review process through a sequential, step-by-step method to formulating feedback as directed by their domain knowledge [39].

Qualitative descriptions offering valuable experience-based context to perceiving why errors are considered a problem for a reviewer is but one facet of understanding barriers to interface usability. The identification of explicit errors can highlight critical interface areas impeding end-user interaction. For example, the similarities and differences in the types, frequency and severity of errors detected by each group were explored to map strengths and perhaps weaknesses when comparing LDs to HCPs and end users.

### 4.3. Error Identification

Meta-aggregation and applying quantitative logic to the analysis highlighted errors in the interface that were shared or discrete to reviewer groups. Experts, on average, were equally skilled at identifying high-frequency content errors; however, as reviewers were evaluating the Toolkit interface, there was a trend for LDs to be more sensitive to errors affecting the user experience of the information. Errors were more likely associated with information flow between and within pages, navigation devices or scripted hyperlink text, and interactions between the site and the user. Typical usability error types were more pronounced within the interface for LDs; HCPs identified a sub-set of these error types, perhaps reflecting common issues that they, as typical users, had previously experienced during their interactions with online technologies. HCPs and LDs identified examples of all categorised error types. As a reflection of their usability knowledge and professional practise, LDs detected four discrete error types that can improve interactions for users who face barriers to using or accessing health information, including visual representations, utility, error recovery, and accessibility.

As a single reviewer group, LDs demonstrated the ability to detect errors at a greater frequency than HCPs and carers whilst, on average, having improved efficiency in identifying errors per evaluator. Rates of error identification across the interface of the CarerHelp prototype indicated that LDs detected similar error quotients as heuristic double expert evaluators [46] and, in some cases, in greater percentages [42,47,48]. This pattern was not observed for HCPs, identifying a relatively low error rate [48] across the interface compared to double heuristic experts. It was negated when combined as an ‘expert’ group identifying over 80% of all errors within the prototype when rates compared to carers, a pattern observed in health [49,50], non-health focused interfaces [27,51] and other research studies [52]. Outcomes from the data analysis suggested that LDs and USERs are more likely to identify similar errors, having identified a higher number of shared error types than the frequency of shared errors within the HCP and USER groups. Additionally, findings indicate the commonality between LDs and USERs in how the information within the user interface is perceived, understood, or comprehended within the context of being a non-specialist in the palliative care domain. It is essential to acknowledge that USERs identified equivalent error types as the expert reviewers, although at a lower frequency.

Study observations and qualitative narratives suggest LDs are well suited to review online health toolkits due to technical skills and awareness of building within online platforms or programs, integrating activities to create interactive experiences, and understanding interface features contributing to functionality, including navigation.

Conceptually, online health toolkit interfaces [43,53] are like those produced by LDs in typical practise within higher education settings. Educational online course materials and toolkits share the requirements for developers to recognise the needs and abilities of learners through interface design. LDs and toolkit developers are observed adopting strategies to translate knowledge through instructional components or interactive features to support learning objectives.

### 4.4. Potential Role of Learning Designers in Usability Evaluations

The combination of HCPs and LDs could effectively generate usability feedback where heuristic double experts are absent or, in many cases, unavailable. Both reviewer groups balanced the types of feedback provided to developers and the variety of error types identified within the interface. The data indicated that each group complemented the other, especially in covering the deficits in identifying errors that are unsighted or not recognised by one group tend to be detected by the other. LDs bring their expertise, knowledge of interaction design and, although usability evaluations are not a standard component of their everyday practise, an awareness of usability principles and their personal ‘baggage’ [54]. An individual’s baggage is linked to life experiences, socio-cultural characteristics shaping interactions online, and the ability to contextualise information to previous lived experiences or empathise with other people’s perceived situations. For this palliative care resource, LDs demonstrated similar interactions with the interface information as the carers, identifying co-existing errors within the interface and offering revisions to content contextualised to the carer. Participating LDs expressed both a personal or shared experience of palliative care/caring for a loved one (voiced from the shared perspective of a family member, friend, or colleague), articulated empathy and voiced a personal connection to someone they know could be caring soon. HCPs were defined by their relationship to the Toolkit content or knowledge and were not forthcoming with a personal perspective. Instead, like their feedback on the usefulness of the Toolkit post-release [55], their feedback was solely from a professional perspective and experience of caring for palliative patients and their carers.

The necessary recruitment of representatives for both review groups to participate in the process was equally problematic. Reviewers, regardless of the profession, were enthusiastic about being involved and whilst remote facilitation enabled involvement geographically, difficulties identifying suitable and available professionals were further compounded by complex development processes.

### 4.5. Can Expert Evaluators Replace End Users in the Development of Digital Health Resources?

For development teams designing and building online health information resources, these two groups of experts could replace users within the evaluation process, as experts are more adept at identifying errors across both skills, technical [26] and rule-based interactions than users [47]. The data also highlighted the dangers of employing expert-only reviewers to undertake usability evaluations in place of end users. Unlike heuristic evaluations [42], USERs and experts have identified similar problems within the Toolkit interface during expert peer-review processes, adding complexities for development teams to recognise the difference between complementary and contradictory errors. Unlike complementary errors or errors detected similarly by more than one reviewer group, error contradiction between groups recognises the presence of an error whilst the other does not perceive the same. For developers, error ‘false alarms’ [32] increase concerns about reiterative decisions shaping interfaces without the end-user voice. Contradictive errors complicate decision-making by knowing which are not veritable within the interface, increasing the risk of resolving for one group and creating a new interface error for another. End users (carers in this study context) can offer developers a voice with lived experience of being a carer providing palliative care even with the levels of extreme difficulty experienced in recruiting carers and having the lowest error detection rates overall.

Qualitative narratives provide a powerful mechanism to reiterate the interface, improve the content, and shape interface functionality. This narrative also adds weight to identifying veritable errors, an alternate perspective to expert opinions, and improving usability and experience within the interface.

### 4.6. Study Limitations

It is important to recognise the exploratory nature of this study as this research approach follows user-centred design principles involving methodologies to generate formative data to inform the reiteration of the interface. Pragmatically, the small evaluator sample sizes reflect typical usability evaluation practise. It is acknowledged that on analysis of usability data, small, subtle, or nuanced differences in the between-group comparisons are unlikely to be detected as the sample size was small and statistically underpowered [32,37]. It is important to recognise that this is not an unusual outcome from feasibility or proof-of-concept studies, where small samples indicate the potential value and validate investment in further research. This study was conducted during the development of the CarerHelp Toolkit; access to stable prototypes was aligned to provide timely feedback to the project group to inform reiteration of the interface. As timelines for the two evaluation methodologies did not align, the two reviewer groups, carers (USER) and experts (LD + HCP), did not review the exact version of the prototype. The USER group utilised an earlier version of the Toolkit, whilst both LDs and HCPs evaluated a similar but later version. This version had identical content, although it featured additional embedded interactives and unavailable resources at usability testing with carers on the earlier version. Whilst this limitation could have resulted in an increasing trend between error frequency and quantity of content between the two usability sessions, many of the interactive features were out of the scope of the expert review process. Nonetheless, it is essential to note that the core of the CarerHelp Toolkit remained constant and was evaluated by all three reviewer groups.

### 4.7. Future Research

Outcomes from this study suggest that there is potential value in LDs and HCPs being involved as composite double experts to generate valuable feedback in the development and design of palliative care digital resource.

Further investigation is required to understand the comparative differences or similarities in the type, frequency and severity of the usability errors identified by composite experts and those detected by trained, heuristic double experts. Areas of commonality between the two evaluator groups could assist multidisciplinary development teams in deciding whether investing time and money to either access or train in-house heuristic experts is worthwhile. This is especially so given the low investment and high return of utilising HCPs and LDs to generate valuable feedback to supplement end-user narratives of the lived experience.

Future research could investigate the types of usability information that might support healthcare professionals to become heuristic double experts. In addition, there is scope to develop, implement and evaluate education or practise guidance that could support healthcare professionals to rapidly expand and develop their existing technical abilities to include scholarship in user-centred design principles and an understanding of the need to consider end-user experience in interface design. The question of composite expert diversity is also an important area to explore, especially given HCPs are both diverse in their abilities, backgrounds, and experiences and in their specialty areas. For example, types of feedback generated by different HCPs such as specialist palliative care physicians and nurses compared to those of generalised specialties who are caring for patient’s clinical and psycho-social symptoms at the end of life. Findings could support the wider application of this approach to develop generalised health online resources or m-health applications supporting health promotion, monitoring, or encouraging behaviour change in the wider population.

## 5. Conclusions

Recruiting end users is difficult, and for multidisciplinary teams creating digital resources for carers of palliative care patients is further complicated by the perceived sensitivity of the subject domain, design inexperience, and working within an increasingly complex development environment. Within a palliative care context, usability evaluations involving LDs as expert evaluators were explored to understand their potential to support HCPs in shaping health interface designs when end users are difficult to recruit. Comparative analysis of content-specific errors found that HCPs were more likely to offer feedback by applying process knowledge to their understanding of the palliative care interface information and carers as end users. LDs identified similar content errors; however, they could offer narratives from their perspective of someone who has experienced death or a loved one dying and were equally adept at identifying high error frequencies. Importantly, LDs were increasingly sensitive to errors impacting end users who face barriers to accessing and using digital health information.

Our research suggests that through their professional aptitude as digital interaction designers and ability to reflect on their lived experiences, LDs have and can provide a unique and valuable perspective as evaluators during usability evaluations. However, feedback generated from carers as end users was highly contextualised to their needs and reflected their experiences as patients, carers or families interacting with healthcare, information, or systems. For developers of digital health interfaces, the involvement of experts cannot replace the insights of the lived experience and the influence of diversity of user characteristics on the types of feedback generated by end-user participation. Recruiting end users for usability evaluations remains a crucial step in creating meaningful and useful interactive experiences whose designs can support our communities’ health information needs.

## Figures and Tables

**Table 1:** Content-specific error descriptors applied to feedback generated by expert review groups (modified Sayoran 1992 [39]).

Content Error Descriptor	Definition of Error Descriptor
1. Evaluation	Positive or negative comments from reviewers, judgements, or preferences
2. Grammatical	Spelling or grammatical corrections
3. Knowledge Statement	Problem with specific content knowledge
4. Problem Identification	Explicit reference to an issue or problems
5. Resources/Activities	Explicit reference to embedded resources and learning activities
6. Revision Statement	Explicit verbalisation or text statement with the intent to change the current to an ideal state
7. Strategies:	Explicit reference to underlying strategies or the need to apply strategies to the content
8. Text Knowledge	Comments or statements from reviewers on learnings from the text

**Table 2:** Demographics and characteristics of healthcare professionals (HCPs) as subject-matter (content) ‘expert’ evaluators assessing the CarerHelp Toolkit prototype.

Professional Position	Practise Setting	Post-Qual. Exp. (Years) ^1^	S-R Tech. Ability ^2^
Nurse/ Director of Service	Acute and Community Care	14	Expert
General Practitioner/ Director	Acute and Community Care	20	Intermediate
Social Worker	Acute and Community Care	7	Expert
Nurse Practitioner	Community Care	10	Expert

^1^ Post-Qual. = Number of years practising after professional qualification to practise was awarded. ^2^ S-R TA = self-rated technical ability: (a) Avoider of everything online—you would prefer to find a ‘real’ person to help; (b) Novice or learner or beginner; (c) Intermediate skills who is mostly confident; or (d) Expert who is confident in finding and using online information.

**Table 3:** Demographics and characteristics of Learning Designers * (LDs) as ‘Digital’ (interaction) ‘expert’ working in higher education to design digital learning experiences.

Professional Position	Post-Qual. Exp. ^1^ (Years)	S-A Tech. Ability ^2^
Learning Designer	27	Expert
Educational Designer	15	Expert
Educational Technologist	12	Expert
Educational Designer	20	Expert
Learning Designer	3	Expert
Learning Designer	5	Expert
Educational Designer	12	Expert

* Learning Designers: Professionals in designing and implementing online educational materials employed in the higher education sector. Other standard equivalent professional titles include Instructional Designer or Educational Designer with Educational Technologist working between educational/learning design tasks and deployment within technological systems or platforms. ^1^ Post-Qual. Exp. = Number of years practising after professional qualification to practise was awarded. ^2^ S-R TA = Self-Rated Technical Ability: (a) Avoider of everything online—you would prefer to find a ‘real’ person to help; (b) Novice or learner or beginner; (c) Intermediate skills who is mostly confident; or (d) Expert who is confident in finding and using online information.

## Data Availability

Data are contained within the article, Appendix A, or Supplementary Material. The data presented in this study are available in the associated Appendix A and Supplementary documentation.

## Supplementary Materials

The following supporting information can be downloaded at: https://www.mdpi.com/article/10.3390/ijerph20054608/s1, (1) Data Collection form—Expert Review and (2) Report—Formal Findings from the Usability Testing of the CarerHelp Toolkit Prototype.

## Appendix A. Data Tables

**Table A1:** Error frequencies identified by Healthcare Professionals and Learning Designers within the prototype.

		HCP Experience (Years)			S-R TA			LD Experience (Years)				S-R TA	
		**6–10**	**11–15**	**16–20**	**Int**	**Exp**	**Total**	**1–5**	**11–15**	**16–20**	**>21**	**Expert**	**Total**
		***n* = 2**	***n* = 1**	***n* = 1**	***n* = 1**	***n* = 3**	**77**	***n* = 2**	***n* = 3**	***n* = 1**	***n* = 1**	***n* = 7**	**202**
**Errors Identified (%Total)**		44 (57.1)	17 (22.1)	16 (20.8)	16 (20.8)	61 (79.2)		54 (26.7)	44 (14.7)	71 (35.2)	33 (16.3)	202 (100)	
**Ave error/user**		22	17	16	16	20.3	**Total (%)**	27	22	71	33	28.86	**Total (%)**
**Type of Error**	Accessibility	0	0	0	0	0	0	0	0	1	2	3	3 (1.5)
	Inform architect.	3	0	1	1	3	4 (5.2)	1	2	0	2	5	5 (2.5)
	Inform flow	6	1	2	2	7	9 (11.7)	15	9	7	6	37	37 (18.3)
	Interaction	0	0	1	1	0	1 (1.3)	2	2	5	0	9	9 (4.5)
	Navigation	8	3	1	1	11	12 (15.6)	7	8	12	2	29	29 (14.4)
	Pedagogy	2	0	0	0	2	2 (2.6)	0	0	1	0	1	1 (0.5)
	Recovery	0	0	0	0	0	0	0	0	2	4	6	6 (3.0)
	Site platform	1	2	0	0	3	3 (3.9)	8	6	10	1	25	25 (12.4)
	Specific content	22	11	10	10	33	43 (55.9)	18	16	28	15	77	77 (38.1)
	Utility	2	0	1	1	2	3 (3.9)	2	0	4	0	6	6 (3.0)
	Visual Repres.	0	0	0	0	0	0	1	1	1	1	4	4 (2.0)
**Nielsen’s Severity Rating**	High (1)	0	0	0	0	0	0	2	5	5	2	14	14 (6.93)
	High-Med (1–2)	4	1	1	1	5	6 (7.8)	0	1	0	2	3	3 (1.49)
	Medium (2)	15	2	3	3	17	20 (26.0)	14	18	24	12	68	68 (33.7)
	Med-Low (2–3)	9	6	7	7	15	22 (28.6)	3	6	10	2	21	21 (10.4)
	Low (3)	16	8	5	5	24	29 (37.7)	35	14	32	15	96	96 (47.52)
**Area of toolkit**	Site	4	0	1	1	4	5 (6.5)	2	2	6	3	13	13 (6.4)
	Menu	0	0	0	0	0	0	2	0	2	0	4	4 (2.0)
	Home	6	1	3	3	7	10 (13.0)	7	8	11	7	33	33 (16.3)
	Carer Pathway	6	5	2	2	11	13 (16.9)	12	7	8	5	32	32 (15.8)
	Being Prepared	3	3	0	0	6	6 (7.8)	2	1	4	3	10	10 (5.0)
	Being a Carer	0	0	0	0	0	0	1	0	0	0	1	1 (0.5)
	Being EoL Carer	3	0	2	2	3	5 (6.5)	5	4	7	2	18	18 (8.9)
	Caring for Dying	1	0	3	3	1	4 (5.2)	4	1	4	3	12	12 (5.9)
	Learning Module	8	3	2	2	11	13 (16.9)	7	10	17	2	36	36 (17.8)
	After Caring	4	3	0	0	7	7 (9.1)	3	1	2	1	7	7 (3.5)
	Carer Library	6	0	3	3	6	9 (11.7)	1	6	5	4	16	16 (7.9)
	Carer Voice	1	2	0	0	3	3 (3.9)	6	4	5	2	17	17 (8.4)
	About Project	2	0	0	0	2	2 (2.6)	2	0	0	1	3	3 (1.5)

S-R TA = Self-Assessment of Technical Ability: (a) Avoider of everything online—you would prefer to find a ‘real’ person to help; (b) Novice or Learner or Beginner; (c) INT = Having Intermediate skills who is mostly confident; (d) EXP = Expert who is confident in finding and using online information HCP = healthcare professional; LD = Learning Designer; Visual Repres. = Visual Representation; Inform architect. = Information architecture.

**Table A2:** Content-specific error examples and frequency of errors identified by expert review groups (HCP and LD).

	Content Errors Identified		
Content Error Groups: Error Definition/Examples	HCP (*n* = 4)	LD (*n* = 7)	Total (%)
**1. Evaluation: Positive or negative comments, judgements, or preferences** “I don’t like the statement ‘caring for someone dying is a major task’” [ERPC4] “Way too much information and duplication … by this time I have given up as it feels like a maze” [ERLD5]	6	6	12 (10.0)
**2. Grammatical: Spelling or grammatical corrections** “People often provide care when someone is older, seriously or has a disability. Think the work ill is missing from seriously” [ERPC2] “Last sentence ‘provide’ should be ‘provided’” [ERLD3]	3	8	11 (9.17)
**3. Knowledge statement problem with specific content knowledge** “Not sure that ‘caring for someone who is dying is the end of a journey of caring’. The dying part is the most intense and most profound and this statement has it over with before the experience has concluded. I would focus on the profound elements of caring for someone dying not the end of the caring role” [ERPC2] “Our other modules on about slide four but they are not consistent. Why are some listed in a module but not in others? I may worry I am missing information I need to know?” [ERLD7]	3	1	4 (3.33)
**4. Problem identification: Explicit reference to an issue or problem** “I think language is okay, but there are just too many words” [ERPC4] “…appropriate to also insert a link here to take the users back to the first page, rather than telling them to go to and use the menu (where is that?) to get back to the main page.” [ERLD4]	8	12	20 (16.67)
**5. Resources and activities: Explicit reference to embedded resources or learning activities** “Need to make sure that this toolkit provides information for carers on how to improve and sustain person’s quality of life when at home” [ERPC4] “CarerHelp Sheets and Videos may require some description because they are specific to the site …not readily apparent what these maybe” [ERLD4]	9	13	22 (18.33)
**6. Revision statement: Explicit text statement with the intent to change current to an ideal state** “Dying is poorly recognised generally. I think it should be assumed that people using it are seeking assistance for a dying loved one. Maybe a reference that dying can occur over a period of time and is characterised by consistent deterioration would be better upfront…If the person you are caring for is dying, then this resource will help you to prepare for the likely changes that will occur in the future.” [ERPC2] “Caring for someone who is dying is the end of a journey of caring …—could be something like: ‘Caring for someone dying also means that your role of carer will come to an end after the person has died. These resources help you be prepared for dealing with the end of life care” [ERLD5]	1	21	22 (18.33)
**7. Strategies: Explicit reference to underlying strategies or need to apply strategies to content** “Not enough information on how this toolkit will help carers—carers will ask, how is this going to help me?” [ERPC3] “… my conclusion is that the Carer Pathways is the entry point that links off to everything else. Maybe these needs explaining more as the starting point, and if you’re returning to the site, you can use the other menus to navigate if you know where you want to go.” [ERLD7]	4	9	13 (10.83)
**8. Text knowledge: Comments or statements from reviewers on learnings from the text** “There are too many words on this page – I don’t think carers will like being told how to feel…” [ERPC4] “… “You might care for a short time or for a long time” could also mean care in the context of how long you personally ‘care’ about the situation rather than the length of time you may have to provide a level of care.” [ERLD1]	9	7	16 (13.33)
**Total (%Total)**	43 (35.8)	77 (64.2)	120
**Mean error/reviewer (*SD*)**	10.8 (3.1)	11.0 (5.9)	

HCP = Healthcare professional, LD = Learning Designer.

**Table A3:** Frequency of overall and unique errors identified by carers (USER) and EXPERT (HCP and LD) evaluator groups.

	Errors Identified by Reviewer Groups							
	HCP (*n* = 4)		LDs (*n* = 7)		USE (*n* = 6)		Experts * (*n* = 11)	
Error Type	^#^ Unique	ˇ Overall	^#^ Unique	ˇ Overall	^#^ Unique	ˇ Overall	^#^ Unique	ˇ Overall
Accessibility	0	0	3	3	2	2	3	3
Information architecture	4	4	3	5	0	0	5	9
Information flow	6	9	24	37	4	9	28	46
Interaction	1	1	6	9	0	0	7	10
Navigation	8	12	17	29	4	16	23	41
Pedagogy	2	2	1	1	0	0	2	3
Recovery	0	0	3	6	0	0	3	6
Site platform	3	3	17	25	2	6	19	28
Specific content	16	43	28	77	6	21	37	120
Utility	2	3	2	6	0	0	3	9
Visual representation	0	0	3	4	0	0	3	4
**Total errors**	42	77	107	202	18	54	133	279
^ **%Total—Unique [*N* = 167]**	25.15		64.07		10.78		88.08 ^^^	
^ **%Total—Overall errors [*N* = 333]**		23.12		60.66		16.22		83.78
**Mean error by reviewer**	10.50	19.25	15.29	28.86	3.00	9.00	12.09	25.36
**Mean error across interface**	3.82	7.00	9.73	18.36	1.64	4.91	12.09	25.36

HCP = Healthcare Professional, LD = Learning Designer, USE = User (Carers). * Expert Reviewer Group are the collective of LDs (*n* = 7) and HCP (*n* = 4) participants. ^#^ U = Unique errors are exclusive within the interface and refer to an exact problem that can be a source of amalgamation of similar errors within the toolkit. ˇ O = Overall errors are all issues or problems identified within the interface and do not consider exclusivity of occurrence. ^ %Total calculated for Expert group *N* = 151 unique errors within the interface for this group when combined.

**Table A4:** Exclusive and mutually inclusive interface errors mapped across and between evaluator groups.

	Interface Errors Identified by Reviewer Groups													
	HCP ˇ (*n* = 4)		LD ˇ (*n* = 7)		USE ˇ (*n* = 6)		HCP + LD * (*n* = 11)		HCP + USE * (*n* = 10)		LD + USE * (*n* = 13)		HCP + LD + USE * (*n* = 17)	
**Interface Area**	Unique	Overall	Unique	Overall	Unique	Overall	Unique	Overall	Unique	Overall	Unique	Overall	Unique	Overall
Accessibility	0	0	3	3	0	0	0	0	0	0	2	2	0	0
Information architecture	2	2	1	1	0	0	2	6	0	0	0	0	0	0
Information flow	4	5	20	25	3	3	2	14	0	0	2	8	0	0
Interaction	1	1	5	8	0	0	1	1	0	0	0	0	0	0
Navigation	4	6	13	20	1	3	3	8	1	7	2	13	0	0
Pedagogy	1	1	0	0	0	0	1	2	0	0	0	0	0	0
Recovery	0	0	3	6	0	0	0	0	0	0	0	0	0	0
Site platform	2	2	12	18	0	0	1	2	0	0	4	12	0	0
Specific content	8	10	20	33	4	5	7	65	1	14	0	0	1	14
Utility	1	1	1	4	0	0	2	4	0	0	0	0	0	0
Visual representation	0	0	3	4	0	0	0	0	0	0	0	0	0	0
**Total errors**	23	28	81	122	8	11	19	102	2	21	10	35	1	14
**% Total—Unique [*N* = 143]**	16.08		56.64		5.59		13.29		1.40		6.99		0.70	
**%Total—Overall errors [*N* = 333]**		8.41		36.64		3.30		30.63		6.31		10.51		4.20
**Mean error/reviewer**	5.75	7.00	11.57	17.43	1.33	1.83	1.73	9.27	0.20	2.10	0.77	2.69	0.06	0.82
**Mean error/interface**	2.09	2.55	7.36	11.09	0.73	1.00	1.73	9.27	0.18	1.91	0.91	3.18	0.09	1.27

HCP = Healthcare Professional, LD = Learning Designer, USE = User (Carers). ˇ Number of exclusive errors identified by reviewer groups which are unique to each group. * Number of mutually inclusive errors identified by more than one reviewer group; these errors are unique to each group of reviewers.
