# Supplementary material for: Learning Designers as Expert Evaluators of Usability: Understanding Their Potential Contribution to Improving the Universality of Interface Design for Health Resources
Source: Int J Environ Res Public Health. 2023 Mar 5;20(5):4608. doi: 10.3390/ijerph20054608 (PMC10001568; doi:10.3390/ijerph20054608)
Supplement: Supplementary file 1 [file ijerph-20-04608-s001.zip › Supplementary File S1- Expert Review Data Collection Tool.pdf]

<u>Supplementary Document: Expert review data collection tool</u>

Section 1. Expert Review Feedback form – The Carers Toolkit Prototype

These questions are to guide you with your review of the Toolkit.
Please provide as much or as little feedback as required, please indicate 'N/A' for those questions that you think do not require any comment.
Also, please <u>do not</u> feel as if you have to answer *every* question.

You are welcome to complete document as you work or alternatively print it out to write notes as you go. This document will form the basis of your evaluation interview that will be scheduled after you have completed the online review. If possible, I would appreciate a copy of this completed document to assist in completing my analysis of this evaluation process.

Thank you again for providing your valuable feedback.

Section 1 : The Carer Toolkit Home Page

After you have had a look at the Home Page could you please provide some feedback on the this page:

1.1 Is the language used appropriate and easily understood?

1.2 Does the text provide enough depth on what information is being provided

1.3 Structure of the 4 navigation boxes and supported text
Is this feature intuitive? Does it make it clear what is included in each section?

1.4 Is there any information, images or text that should be on the Home page which is not currently there?

1.5 Do you have any comments on the way that the Home Page is structured?
Did you easily find the menu and search functions?
This could be in relation to content, menus or navigation structures (buttons or icons), how the information flows on the page, how it looks visually or how this page makes you feel?

1.6 Do you like or dislike this page? Were there any issues or problems?

Section 2. Content Pages

We would like you now look at the content pages of the Toolkit.

Once you have had an opportunity to navigate through these pages, please reflect on your experience using the Carer Pathway page.

2A) *Carer Pathway*

2A.1 What do you think of the content descriptors for each of the pathway sections?
Are they descriptive enough, is the language appropriate and is the tone correct?

2A.2 Do you think this is a useful page to orient carers to their current position within the caring trajectory?

2A.3 Is this page something that carers will identify with?

2A.4 Is this page a good way to move into the other content pages of the Toolkit?

2A.5 Do you like or dislike this page? Were there any issues or problems?

Once you have found your way to the other content pages, please have a look at these four pages and provide some feedback on some of the following aspects.

2B) *Being an EoL Carer*

2B.1 The information is structured on each page – is everything in the right place?

2B.2 Is the information on the page easy to understand, do the hyperlinks take you to the place you thought you might be going?

2B.3 Is there information that you would consider to be missing from the pages?

2B.4 Is the language and tone correct for this content in context for each point in the carer's trajectory?

2C) *Being Prepared*

2C.1 The information is structured on each page – is everything in the right place?

2C.2 Is the information on the page easy to understand, do the hyperlinks take you to the place you thought you might be going?

2C.3 Is there information that you would consider to be missing from the pages?

2C.4 Is the language and tone correct for this content in context for each point in the carer's trajectory?

2D) *Caring for the Dying?*

2D.1 The information is structured on each page – is everything in the right place?

2D.2 Is the information on the page easy to understand, do the hyperlinks take you to the place you thought you might be going?

2D.3 Is there information that you would consider to be missing from the pages?

2D.4 Is the language and tone correct for this content in context for each point in the carer's trajectory?

2E) *After Caring*

2E.1 The information is structured on each page – is everything in the right place?

2E.2 Is the information on the page easy to understand, do the hyperlinks take you to the place you thought you might be going?

2E.3 Is there information that you would consider to be missing from the pages?

2E.4 Is the language and tone correct for this content in context for each point in the carer's trajectory?

2F) *Overall feedback on the Content pages*

2F.1 After viewing the pages, do you think the learning modules a useful tool for providing information to carers?

2F.2 Do you think carers will use them, if so in what context?

2F.3 What did you like or dislike about these pages? Was there any issues or problems?

Section 3. Carer Voice

After having a look this page, we would like you to provide some feedback:

3.1 Do you think that these videos will be useful and relevant to carers?

3.2 Do you think that carers will view these videos and will they provide support?

3.3 Is there enough information on the page to help carers know what they are viewing

3.4 Do you like or dislike this page? Were there any issues or problems?

Section 4. Carer Library

As a place for many different resources, could you please review this page and tell us if:

4.1 The information is organised in a way that assists you to find specific information easily?

4.2 Are the section headers providing enough of a description that what you find is what you expect in each section of the resources on this page?

4.3 Is there any information or resources missing from this page in your view as a health care professional?

4.4 Do you like or dislike this page? Were there any issues or problems?

Section 5. About the Project

5.1 After viewing this page, is there any information that you think should be added to provide carers with an increased sense of trustworthiness or indicators of quality?

Section 6. General Feedback on the Carers Toolkit

After you have had a chance to look though and interact with The Toolkit, we would appreciate your feedback on the following :

6.1 Do you think that the way that the Toolkit is structured and the content, types of activities and resources fits together to provide a positive experience for carers?

6.2 Do you think this resource is relevant and useful to carers based on the content and structure of the Toolkit?

6.3 Do you think the is content relevant to carers?

6.4 Are there any needs or gaps in the information provided that could be added to the Toolkit to support carers in the community?

6.5 Would you recommend the Carers Toolkit to carers you would support in your clinical practice?

Section 2. Post-Feedback Interview Questions

General discussion: Pinch and Pain points as identified from the feedback form data

- PAIN POINTS - Major problems or issues that you found within the toolkit (break points)
- PINCH POINTS - Minor problems or issues identified (pain points)

Other points of discussion:

1. Content for toolkit - Quality of content provided

a) Do you think that the way the Toolkit is structured and the way that information is presented makes it easy for carers to access this information?

b) Do you think that this toolkit caters to the diversity in technological abilities of carers that will be using this toolkit?

c) What about the diversity of carers as a whole: considering education levels, occupations, socioeconomic status and understanding or comprehension of English language?

2. Language and concepts – too gentle or not hard enough in the descriptions of the roles that carers will undertake and those experiences that they will encounter
3. Should the developers be braver perhaps using harder language or direct descriptions?
4. Will carers be receptive to this type of language?
5. Do you think that carers information searching behaviours have changed and are they seeking out information about the end of life?
