# Supplementary material for: Learning Designers as Expert Evaluators of Usability: Understanding Their Potential Contribution to Improving the Universality of Interface Design for Health Resources
Source: Int J Environ Res Public Health. 2023 Mar 5;20(5):4608. doi: 10.3390/ijerph20054608 (PMC10001568; doi:10.3390/ijerph20054608)
Supplement: Supplementary file 1 [file ijerph-20-04608-s001.zip › Supplementary File S2 - Formal Usability Report - The Carers Toolkit Prototype.pdf]

Report of Usability Test Findings of the Australian Carers Toolkit

Prepared by:
Amanda Adams
Flinders University

26 August 2019

TABLE OF CONTENTS

EXECUTIVE SUMMARY 3
- SUMMARY OF FINDINGS 3
- Strengths 3
- TO BE IMPROVED 3

1. STUDY OUTLINE 4
- 1.1 Test dates: 4
- 1.2 Location: 4
- 1.3 Purpose of test: 4
- 1.4 Areas tested: 4
- 1.5 Test facilitator: 4

2. METHODOLOGY 4
- 2.1 Index of Findings 4
- 2.2 LIST OF FINDINGS 4

3. DETAILED FINDINGS 6
- 3.1 HOME PAGE 6
- 3.2 CONTENT PAGES 9
- 3.3 LEARNING MODULES 11
- 3.4 OTHER FEEDBACK 13
- 3.5 CARER PATHWAY 14
- 3.6 CARER VOICE 15
- 3.7 CARER LIBRARY 16

4. APPENDIX: TECHNICAL REPORT 19
- 4.1 USABILITY SESSION SUMMARY 19
- 4.2 TASK SCENARIOS, TASK CHARACTERISTICS, TASK SUMMARY & PARTICIPANT LIST 19

EXECUTIVE SUMMARY

SUMMARY OF FINDINGS

Overall, the participants found the Carers Toolkit to be a generally useful and relevant online resource that will be acceptable to the targeted audience. Participants found the content to be extremely valuable especially the practical tools and guidance supporting care during the intermediate stages before the terminal phase. The majority of issues identified by this usability evaluation were related to the navigation into and around the internal pages of the Toolkit including text descriptors and landmarks whilst the language used in the protype was highlighted as a problem for some carers.

STRENGTHS

- The way the Toolkit looks provided a pleasant experience to carers with the colours and drawn images being well received particularly in the Carers Pathway to sensitively portray pictorial representations of the stages of caring.
- The structure (menu and information design) of the Toolkit was easily learnt by carers after their initial visits to the site, carers were pleased with the simplicity of the design.
- Resources and tools within the Toolkit was found to be of great relevance to carers and all identified information that would have been helpful during their caring experiences.
- Carers involved in this evaluation all provided positive feedback on the need of the resource, were moderately satisfied with their experience using the prototype and that they would recommend the resource to other carers particularly early in their caring experience.

TO BE IMPROVED

This design does not lead people quickly and easily through the Toolkit to find specific information especially when arriving at the Home Page. In particular:

- It is unclear on how visitors can move from the Home Page into the correct sections of the Toolkit if they are not shown what is in the Toolkit when arriving
- Lack of visible menu or poorly described navigation buttons provides difficulty to older people or people with limited technological skills or experience to recognise not traditional functions of newer designs; this includes the ☰ menu button and icons that change colour when hovering but have no function.
- The steps moving into the Toolkit pages are not clearly described or landmarked especially in relation to the Carers Pathway page
- Key information was missed due to the structure of content on the pages as visitors with poorer technological skills and experience don’t always look everywhere on a page for information
- Balancing the language to be acceptable to all carers regardless of stage of caring will be important in providing a satisfying experience for users

---

DEFINITIONS USED IN THIS REPORT

The Toolkit or prototype: The Carers Toolkit website

Interface: Can describe all information on the screen, the device and associated hardware such as mouse, printers and integration across different software.

1. STUDY OUTLINE

1.1 TEST DATES: 8th – 15th August 2019

1.2 LOCATION: Rehabilitation and Palliative Care Building, Flinders Medical Centre, Adelaide

1.3 PURPOSE OF TEST: Identify usability issues and feasibility of Carers Toolkit prototype to be addressed in the redesign

1.4 AREAS TESTED: General usage of finding information and overall impressions of the Carers Toolkit

1.5 TEST FACILITATOR: Amanda Adams

2. METHODOLOGY

Six people participated in the study. Participants were given tasks to perform and asked to use the think aloud protocol. Test sessions lasted an average of 70 minutes.

2.1 INDEX OF FINDINGS

For easy reference, the findings in this report are identified as **High**, **Med**(ium), **Low**, or ***Good*** and appear as follows:

1. **GOOD: The design was simple and users understood it. Keep it.**
2. **HIGH: The design or feature caused extreme confusion or frustration; problem has large impact on task flow.**
3. **MED: The design or feature caused some confusion.**
4. **LOW: The design or feature caused little confusion or frustration; the feature is not in a high priority area of the site.**

Recommendations look like this. One recommendation may address several usability issues.

Quotes from participants look like this

2.2 LIST OF FINDINGS

1. **HIGH:** It is unclear on what information the Toolkit has to offer new visitors to the site 6
2. **HIGH:** Lack of navigation cues produced indecision in carers trying to locate the correct path into the Toolkit from the Home Page 6
3. **HIGH:** It is difficult to know the sections that are within the Toolkit from the navigation and landmark cues on the Home Page............ 6
4. **MEDIUM:** A visible search function on the Home Page would decrease the level of frustration observed when trying to find specific information in Toolkit sections 7

5. MEDIUM – LOW: Quality indicators are hidden below the fold and more information on the HP about how carers contributed to the process of development could increase users sense of authenticity and provide reassurance that this information is relevant 8
6. HIGH: 'Key Resources' were commonly missed during visits to these content pages as participants did not scan below the fold 9
7. HIGH: Language within these pages were found to be impersonal and patronising that provided a frustrating experience for some carers 9
8. GOOD: 'Our Suggestions to you' provided a mix of activities that can provide information presented in different modes 10
9. HIGH: Content deemed to be important to carers that will assist with caring needs should be prioritised on page position to be in immediate eye line for users to view on opening the page 11
10. HIGH: Language and link descriptors provoked frustrated and irritated reactions from carers to the impersonal and educational approach demonstrated by these descriptions 11
11. GOOD: Inclusion of learning modules provide a different style of presenting information providing an experience that will benefit learners who are looking for the 'whole' picture 12
12. LOW: Section headers may need to be reviewed as these were found to be non-descript 13
13. MEDIUM-HIGH: Additional information on legal issues and palliative care identified as some key information needed in the Toolkit that is currently missing 13
14. GOOD: Carer Pathway was provided a common sense and straight forward pathway that carers can identify where they are in the caring trajectory 14
15. LOW-MEDIUM: Descriptions within the Carer Pathway caused some confusion as they provided no sense of what a carer will find within each section of the pathway 14
16. MEDIUM: How does the Carer Pathway page fit within the navigation structure/information flow of the Toolkit as there was some confusion of it's role in the website 14
17. GOOD: This format for exploring carers experience was acknowledged as a good option for carers to get the whole picture especially on what to expect. 15
18. HIGH: Descriptors provided no information on what is covered in each video 15
19. LOW-MEDIUM: Need to include information about complex illness and caring journeys for carers as these are unpredictable and don't always follow the caring pathway 15
20. HIGH: Although there were section headers for guidance, there was difficulty in finding information in each of the sections of the library 16
21. MEDIUM: Search function would be helpful to find information in the Carer Library in a more direct and effective manner 16
22. LOW-MEDIUM: Complex illness and caring journeys for carers are unpredictable and don't always follow the caring pathway 17
23. HIGH: Information displayed in the Toolkit was hard to read and light in contrast to the background 18
24. HIGH: Balance in language and tone will be important for this group of users particularly as there will be variation in self-identification of where carers are in the caring trajectory, levels of preparedness-acceptance-lived experience and types of information required based on the level of support being received in the community 18

3. DETAILED FINDINGS

3.1 HOME PAGE (https://www.carerhelp.com.au/tabid/5611/Default.aspx)

1. HIGH: IT IS UNCLEAR ON WHAT INFORMATION THE TOOLKIT HAS TO OFFER NEW VISITORS TO THE SITE

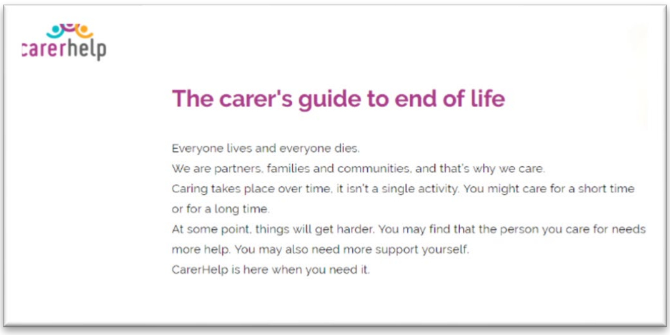

Participants commented that the text on the home page (HP) provided little guidance on the practical guidance that is provided within the toolkit. Over half described the text as “project speak” and provided no real information on carers should go any further into the toolkit, this was noted as being particularly important as this information is in prime view of users arriving at the site.

→ Modify text to include how the toolkit can help carers and include descriptions of practical support that this resource has to offer to help users with decision-making process.

2. HIGH: LACK OF NAVIGATION CUES PRODUCED INDECISION IN CARERS TRYING TO LOCATE THE CORRECT PATH INTO THE TOOLKIT FROM THE HOME PAGE

Carers were moving into the internal sections of the Toolkit from the HP primarily using the four “navigation buttons” at the bottom of the first screen. Observations showed that even though carers knew that either ‘Carers Voice’ or ‘Carer Library’ was not the correct option for finding specific information, they were willing to accept ‘incorrect pathways’ in order to access the internal menu system at the top of the screen of all other pages.
Acceptance of this behaviour should be cautioned due to the conditions of usability testing, as ‘regular’ carers may not be so forgiving when trying to navigate to the correct section via multiple incorrect clicks and leave the site unsatisfied.

→ Need to include brief descriptions in the ‘buttons’ that will give the user more of a sense of what they will find in each section which will provide improved guidance into the Toolkit to decrease the levels of frustration.

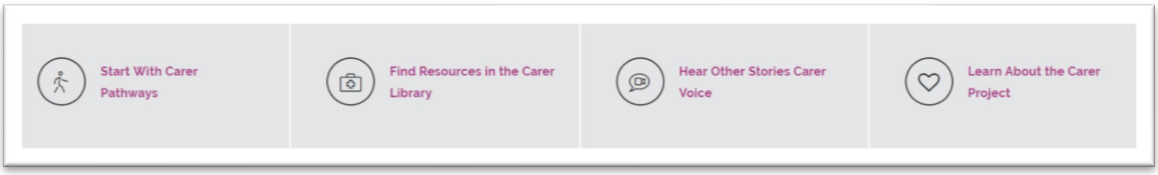

3. HIGH: IT IS DIFFICULT TO KNOW THE SECTIONS THAT ARE WITHIN THE TOOLKIT FROM THE NAVIGATION AND LANDMARK CUES ON THE HOME PAGE

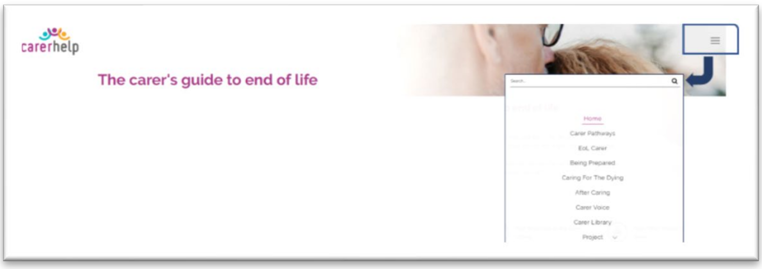

Features on the HP that assist users to formulate a pathway to specific information within the Toolkit include two mechanisms - four ‘navigation buttons’ and a menu associated to the skin of the website (top LH corner – ‘Hamburger’ icon ≡). Participants were found to using the ‘navigation’ buttons the majority of the time to access the internal pages, but these only describe four of the internal sections and do not reference the content sections (Being an EoL Carer, Being Prepared, Caring for the Dying and After Care).

This structure contributes to the incorrect pathways observed (as described above) and increased frustration as carers were often surprised and overwhelmed at the amount of content they found once they got past the HP. Interestingly, only 1 of the participants used the ≡ menu on the HP to navigate into the Toolkit, this carer could be considered an 'expert' in technological skills and abilities and was familiar which this structure due to her browsing on a smart phone. All other participants were shown the ≡ on the HP during the session, but all still used the buttons to navigate into the Toolkit.

*[I am] "more likely to find a search thing and search for a particular word rather than clicking through pages. Cause I would probably get the sh**s clicking through things really quickly. Because when you are stressed out and caring for someone and you are doing all that, the last thing you want to do is to sit here and click click click. I just prefer to have at my fingertips so to speak - I think most people, even having the menu right at the start more visible and easier to access" [would help].*

(Carer 1)

→ Due to the demographics of carers (generally older with intermediate to low levels of technological experience and ability) the ≡ menu will not be readily adopted by users.

→ There is still a need to provide information about what is in the toolkit and the implementation of a visible menu could solve this issue. Where design limitations exist (vertical or horizontal menu are not possible) other visual cues could be implemented including increasing the number of 'navigation buttons' to include the other sections or use an image or text to funnel users to a specific starting point such as the 'Carers Pathway' page.

4. MEDIUM: A VISIBLE SEARCH FUNCTION ON THE HOME PAGE WOULD DECREASE THE LEVEL OF FRUSTRATION OBSERVED WHEN TRYING TO FIND SPECIFIC INFORMATION IN TOOLKIT SECTIONS

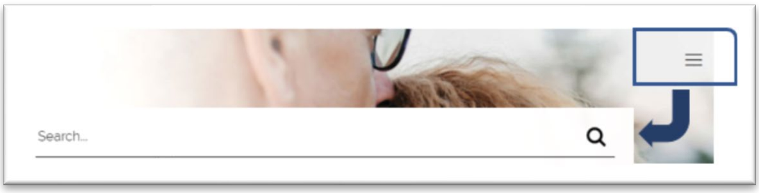

The search field option is "hidden" in the ≡ menu, a feature which majority of participants did not see or did not know what this button did. Participants were asked if this feature was something they were used to seeing, one participant of the six knew what this icon did and used this to navigate into the Toolkit. The remaining five commented the ≡ menu was 'new' to them and that they were used to seeing a structured and visually obvious menu running either horizontally or vertically on the page with drop downs. Users could learn to use this function, but this would take several visits to get used to and this should not be assumed across all users.

→ If the ≡ menu remains the formal navigation and search tool on the Toolkit, then a possible solution would be to include a 'Getting Started' page or 'Hints' section on the Home Page which could help your users to make the transition to this menu type.

5. **MEDIUM – LOW:** QUALITY INDICATORS ARE HIDDEN BELOW THE FOLD AND MORE INFORMATION ON THE HP ABOUT HOW CARERS CONTRIBUTED TO THE PROCESS OF DEVELOPMENT COULD INCREASE USERS SENSE OF AUTHENTICITY AND PROVIDE REASSURANCE THAT THIS INFORMATION IS RELEVANT

Participants were asked to locate indicators of quality and trust within the Toolkit. All six located the institutional logos at the bottom of the page also the statement of funding. Only half of the participants located the 'About the Project' page and thought that this was valuable information should be included on the HP.
All participants verbalised the importance of knowing that this Toolkit has been developed and evaluated in co-operation with carers and thought this informaiton could provide an increased sense of reassurance that content is written from the point of view of the carer.

"I would think on the very first page there needs to a background on who was involved in how this was developed, which would provide greater indication to people that it is trustworthy and has been researched properly. Not something that has just been made up off of the top of someone's head".

(Carer 2)

[This] "Would be reassuring, that actual people on the ground level had been involved".

(Carer 4)

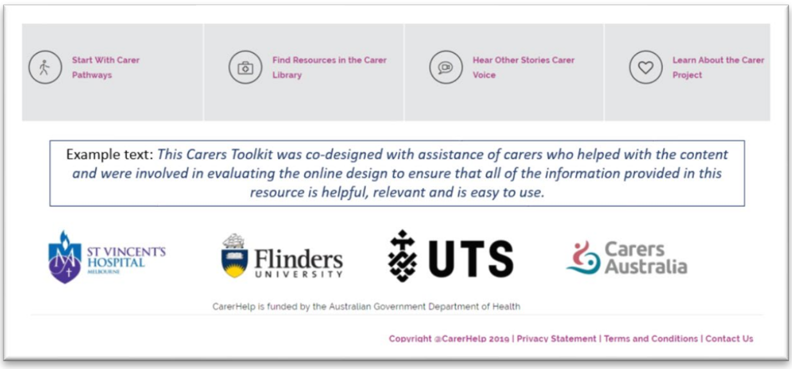

→ Add a sentence to the Home Page perhaps just below the navigation buttons that includes a statement that cares were involved in a participatory relationship with the project team to develop the content and to assist with the evaluation of the Toolkit to ensure that the website is easy to use.

3.2 CONTENT PAGES

- Being an EoL Carer (https://www.carerhelp.com.au/tabid/5612/Default.aspx)
- Being Prepared (https://www.carerhelp.com.au/tabid/5613/Default.aspx)
- Caring for the Dying and After Caring (https://www.carerhelp.com.au/tabid/5614/Default.aspx)
- After Caring (https://www.carerhelp.com.au/tabid/5577/Default.aspx)

6. **HIGH:** 'KEY RESOURCES' WERE COMMONLY MISSED DURING VISITS TO THESE CONTENT PAGES AS PARTICIPANTS DID NOT SCAN BELOW THE FOLD

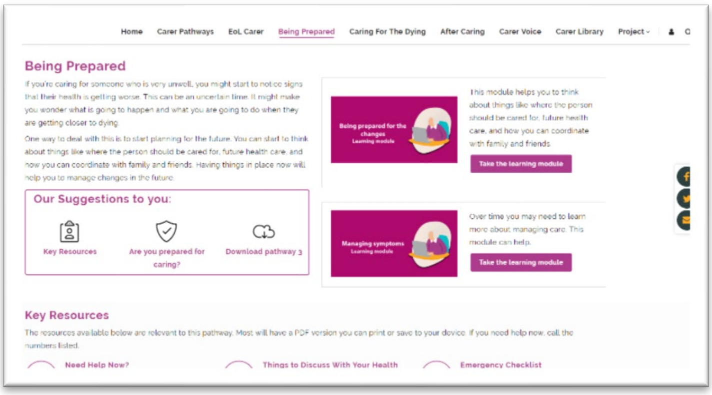

A common trait of participant searching behaviour was observed in the initial tasks undertaken by all carers where content below the fold of the first screen was not viewed. 'Key Resources' were regularly missed especially in the first or second activity when the site was relatively 'new' to the participants. Often this section of the page was discovered by accident, but after the user was aware of the page structure these were regularly views as they were considered the most practical and useful for carers from the content on the page.

> *"I am terrible at scrolling….*
> *This is better [pointing to key resources]. If I was looking for this [Managing Symptoms link] ... This is a practical thing that would be helpful, and resources are great. These are the things you do on a day to day basis. Learning modules may be great to do when you have time".*
>
> *(Carer 6)*

→ Recommend changing information flow of content pages to reflect participants view of 'Key Resources' being at the top of the page (in plain view or immediate line of sight) if this section is considered to reflect the principle objective of the Toolkit. Would suggest moving the 'Key resources' to the position where the learning module sits alongside the descriptor of the purpose and outline of content found on the page.

→ This comment is relevant to the Learning Module section below.

7. **HIGH:** LANGUAGE WITHIN THESE PAGES WERE FOUND TO BE IMPERSONAL AND PATRONISING THAT PROVIDED A FRUSTRATING EXPERIENCE FOR SOME CARERS

The language and tone within these pages will require balance between using realistic and 'hard' language whilst maintaining a personal and supportive tone that will cater for those carers who are going to be an EoL carer in the near future but projecting a realistic and practical tense for carers who are currently "living the experience".

Participants found the text describing the page (top of the page) to be "project speak" and "fluffy" in nature. Comments suggest that this text needs to be harder and alerts carers to the practical information that can be found within each page – that is carers need to know what is in the page for them to use.

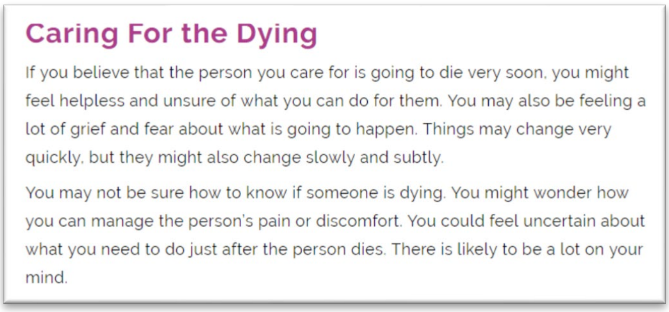

→ Recommend updating text to be less non-descript, that is sharpen the language around the purpose and the content of the page and provide a 'harder' narrative about how this content/resources will help carers at [a specific] stage of caring. Participants suggested not sugar coating the realities of the process particularly around the practical aspects of caring for a loved one who is dying.

8. **GOOD:** 'OUR SUGGESTIONS TO YOU' PROVIDED A MIX OF ACTIVITIES THAT CAN PROVIDE INFORMATION PRESENTED IN DIFFERENT MODES

All participants found the 'Our Suggestions to You' both helpful and useful but indicated that there could be other key resources that could be included in this box perhaps pulled from the 'Key Resources' section.
Although it is noted that descriptors for some of these resources were not detailed enough or specific to what the carer will find when opening the link.

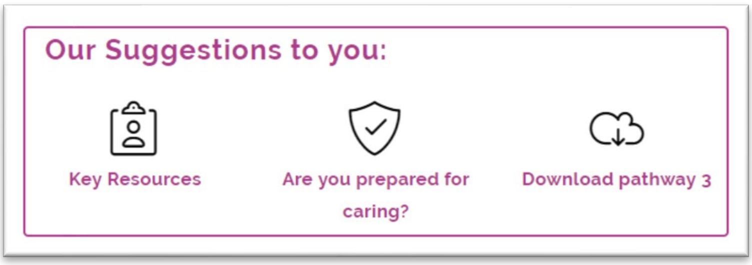

→ Suggest including some information about legal process and access to documents in the 'Our suggestions to you' section on both the 'Being Prepared' and 'Caring for the Dying' sections. Language activity descriptors would also need updating as the use of Take the and Learn this was not well received along with non-precise wording linking activity hyperlink and what is expected when activated.

3.3 LEARNING MODULES

9. **HIGH:** CONTENT DEEMED TO BE IMPORTANT TO CARERS THAT WILL ASSIST WITH CARING NEEDS SHOULD BE PRIORITISED ON PAGE POSITION TO BE IN IMMEDIATE EYE LINE FOR USERS TO VIEW ON OPENING THE PAGE

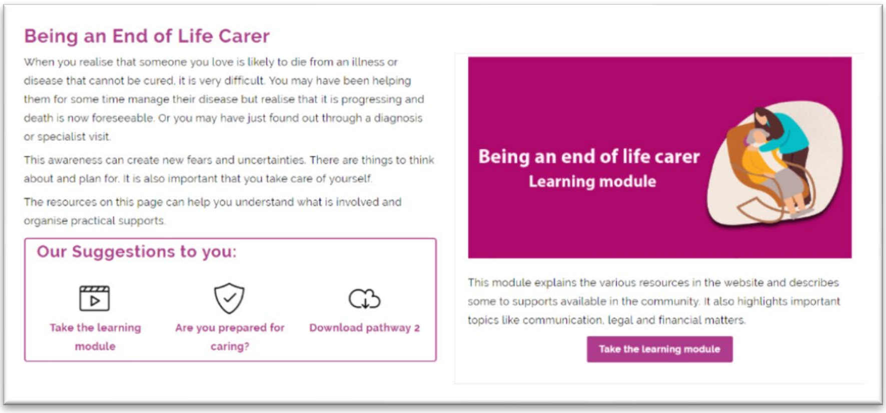

All participants commented that the learning modules were an interesting and useful mode of providing information in a different format contrasting to information in a written or visual format. Caution was advised though that participants would only view these in a 'real-life scenario' if they had time and space to think and 'take in the information'. Realistically, for all these carers, this would be an under-utilised resource as this sample group suggest that they would not use these to access information for practical solutions or support for their caring needs. This contrasted with the 'Key Resources' which all participants found to be relevant, immediately recognised as a useful tool and something that would help them to care at that stage in their journey.

"I think being a carer is more emotional than this. I would do this if this was part of my job and part of the hard bit about caring is that you have already start to loose your relationship with your loved one, if you are a carer, that relationship starts to change. When you are being a carer and not a wife, or a daughter or whatever, I think that this [pointing to the learning module text] really highlights this.
Not very personal, I am not taking a course. I am looking after someone.
This is a personal journey that is already eroding a relationship ... Not breakdown, but the dynamics change and that is part of looking after someone that is dying. And to have things like modules and stuff like that makes even more depersonalising" [experience].

(Carer 6)

→ See comments referring to the 'Key Resources' in the Content section above [Reference section number]

10. **HIGH:** LANGUAGE AND LINK DESCRIPTORS PROVOKED FRUSTRATED AND IRRITATED REACTIONS FROM CARERS TO THE IMPERSONAL AND EDUCATIONAL APPROACH DEMONSTRATED BY THESE DESCRIPTIONS

Descriptive language around the learning modules evoked the highly emotional feedback from over half of the participants. Carers reported that this language – such as "Learn …" or "Take the Learning module" depersonalises their lived experience, as they are not students in a professional learning situation where they will achieve a certificate on completion. This language highlights the painful realisation that their relationship with the person they are caring for has changed. The caring role requires the need for carers to be removed, operating in an almost job-like manner and be devoid of emotion in order to provide the care to their loved ones.

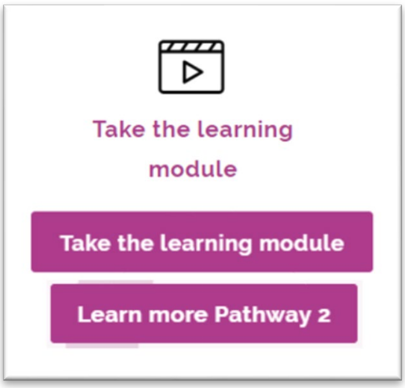

"I am not a student, I am a carer" ………… "Why would I want to learn it when I am living it?"

(Carer 6)

→ Recommend changing the language used within the labels of the text relating to the learning modules on all of the pages, this was a aggravation for over half of the participants and would require use of personal contextual narrative that does not imply nor highlight the difficulties that carers experience. Consider changing these to Information Modules or Information course to remove the unintentional student-teacher dialogue within the Toolkit.

11. GOOD: INCLUSION OF LEARNING MODULES PROVIDE A DIFFERENT STYLE OF PRESENTING INFORMATION PROVIDING AN EXPERIENCE THAT WILL BENEFIT LEARNERS WHO ARE LOOKING FOR THE 'WHOLE' PICTURE

All participants recognised that there would be some carers that would use these modules, but suggested there use will be in relation to time, the type and specifics of the information required, the ability for carers to stop the module and return to where they were up to if they get called away.

3.4 OTHER FEEDBACK

12. LOW: SECTION HEADERS MAY NEED TO BE REVIEWED AS THESE WERE FOUND TO BE NON-DESCRIPT

Examples: ’Being Prepared’ (for what) and Being an EoL Carer (what does EoL stand for?)

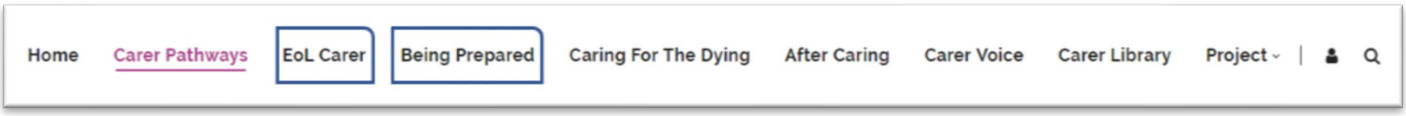

→ Could include a webpage or downloadable PDF that could provide some guidance on the language and features of the Toolkit to assist non-technological users whilst supporting carers who are not health-literate or familiar with medical jargon. This resource could also include hints on the menu and search functions or highlight key tools or resources that could be useful/relevant.

13. MEDIUM-HIGH: ADDITIONAL INFORMATION ON LEGAL ISSUES AND PALLIATIVE CARE IDENTIFIED AS SOME KEY INFORMATION NEEDED IN THE TOOLKIT THAT IS CURRENTLY MISSING

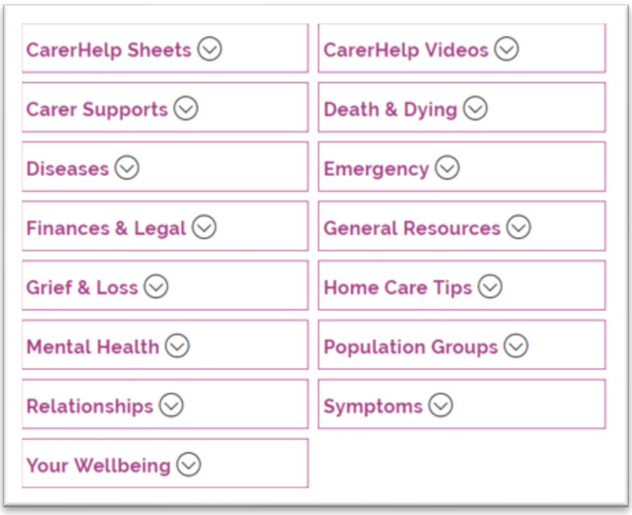

One of the tasks that was asked of the participants was to locate the directory of palliative care services from within the Toolkit, two carers were able find this reference in the Carers Library but all six commented that both the process of accessing and the importance of what palliative care services can offer was extremely important.

Participants were also asked to locate reference to ‘Making Healthcare Decisions for Someone Else’ reference in the Carers Library, in which none of the six were successful. This task lead to discussion on if, why and where information about the legalities of being prepared for dying. All six highly recommended some information be provided within the Toolkit as this issue was one of the more difficult to negotiate as the caring process advanced toward death and that there were serious implications for carers if not initiated early.

→ Palliative care information will be a relevant and important additional information for the Toolkit, recommend inclusion of a PDF or section within the ‘Being Prepared’ providing further information on roles and assistance that palliative care services provide to carers (not from the view of the dying person). The Carer Library page would also benefit from the inclusion of a palliative section with hyperlink to relevant resources

→ Legal information was also highlighted as an information deficiency within the Toolkit. This could sit within the ‘Being Prepared’ key resources or ‘Our suggestions for you’ and be included as a separate section within the Carer Library (already updated).

3.5 CARER PATHWAY (https://www.carerhelp.com.au/tabid/5635/Default.aspx)

14. GOOD: CARER PATHWAY WAS PROVIDED A COMMON SENSE AND STRAIGHT FORWARD PATHWAY THAT CARERS CAN IDENTIFY WHERE THEY ARE IN THE CARING TRAJECTORY

15. LOW-MEDIUM: DESCRIPTIONS WITHIN THE CARER PATHWAY CAUSED SOME CONFUSION AS THEY PROVIDED NO SENSE OF WHAT A CARER WILL FIND WITHIN EACH SECTION OF THE PATHWAY

"All of these pathways descriptions are not very explanatory of what information you are going to be able to see if you open that pathway up - There is nothing here that tells me if there is anything that could help me for practical decisions".

(Carer 6)

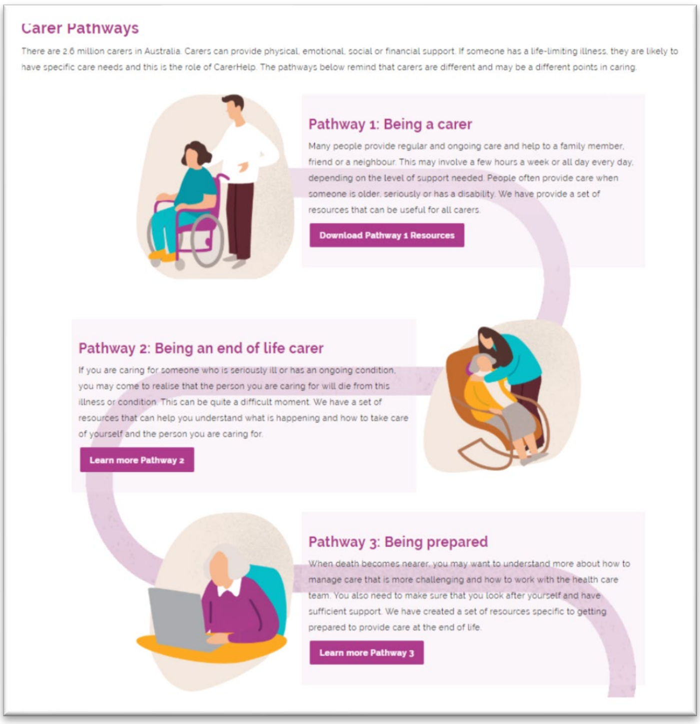

→ Suggest modifying text descriptors for each of the pathways within this page to better describe the information that is found within each pathway and include a brief outline of the practical tools/resources that are found within the pages.

This will enhance users decision-making process to better choose the correct pathway for them whilst providing specific guidance to others on resources or tools available.

16. MEDIUM: HOW DOES THE CARER PATHWAY PAGE FIT WITHIN THE NAVIGATION STRUCTURE/INFORMATION FLOW OF THE TOOLKIT AS THERE WAS SOME CONFUSION OF IT'S ROLE IN THE WEBSITE

All participants found this page to be useful but it was observed that there was a disconnection in where and how the users integrate this pathway into their interactive experience. There was some uncertainty to what they were supposed do once they landed on this page from the HP.

→ Recommend adding some narrative on the Home Page that describes how to use the Carer Pathway especially if this is going to be used as a 'gateway' page influencing the flow of information and user paths through the Toolkit.

> In the Carer Pathways page, we highlight different pathways of caring for someone with a life-limiting illness. There are a range of general resources that are useful for all carers which we have created as Pathway 1: Being a Carer. The other pathways can be accessed via the Carer Pathways page of via the main menu.

This could also be added to the 'Getting Started' webpage/PDF if implemented [there is some information in the Carer Library which accurately describes it's function]

3.6 CARER VOICE (https://www.carerhelp.com.au/tabid/5749/Default.aspx)

17. GOOD: THIS FORMAT FOR EXPLORING CARERS EXPERIENCE WAS ACKNOWLEDGED AS A GOOD OPTION FOR CARERS TO GET THE WHOLE PICTURE ESPECIALLY ON WHAT TO EXPECT.

Many carers explained that whilst this was a good option for some carers particularly for those starting out on the carers journey, this would be a resource that they may find very useful and inciteful to support expectations. Two thirds of the carers undertaking evaluations said in no uncertain terms that they would not view these as they do not want to hear about other people experiences as they have both enough to deal with and watching these would provide little comfort to their caring experience.

18. HIGH: DESCRIPTORS PROVIDED NO INFORMATION ON WHAT IS COVERED IN EACH VIDEO

Participants commented that they would like to know what type of information could be found in the videos, this was important particularly when time is an issue. A short description of the topics covered in each of the video will enhance the users decision-making processes as to which video to see and reduce the chance of wasting time on topics which are not needed.

→ Text descriptors will be crucial to guide users on which video will suit users needs and provide a sense of reassurance that carers could systematically view these in a manner that could build on their knowledge or support the expectations depending where in the caring trajectory they are.

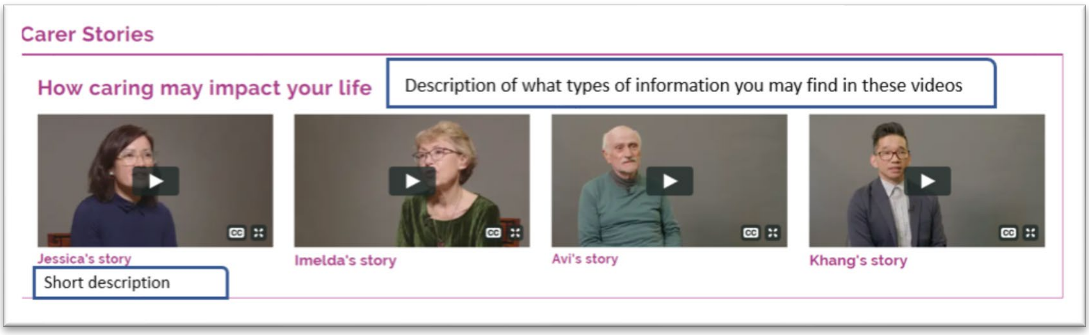

19. LOW-MEDIUM: NEED TO INCLUDE INFORMATION ABOUT COMPLEX ILLNESS AND CARING JOURNEYS FOR CARERS AS THESE ARE UNPREDICTABLE AND DON'T ALWAYS FOLLOW THE CARING PATHWAY

Two of the participants had experienced caring for loved ones with complex illnesses toward the end of their lives and found there was a lack of information for carers in this not uncommon situation. There were suggestions of additional resources that would provide some support and comfort to carers in this situation as there is a lack of information in the wider palliative care sector particularly around uncertainty and the impacts on carers.

"The thing I find really difficult is that my husband is not the normal case …. So I don't know whether to prepare myself and I need a large hospital bed in my lounge room or whether that is the course we will take ... I don't know which path. For me it is not a normal pathway that he has got ... This is interesting information, but I just don't know what is in front of me and is what I find most difficult is that I can't plan".

Carer 5

→ An option to include information for those carers who don't necessarily fall into the pathway categories would be of great assistance especially as there is limited information to be found for carers and provided by health professionals. Therefore, there is a high likelihood that these carers will actively be seeking specific information on what to expect and what to do.

3.7 CARER LIBRARY (https://www.carerhelp.com.au/tabid/5615/Default.aspx)

20. HIGH: ALTHOUGH THERE WERE SECTION HEADERS FOR GUIDANCE, THERE WAS DIFFICULTY IN FINDING INFORMATION IN EACH OF THE SECTIONS OF THE LIBRARY

All participants found their experience within the Carers Library as being “muddled”, “overwhelming” and “confusing” with all carers making use of the section headers to navigate down the page to the identified group of resources. They still were unable to find the required information, spending immense time randomly browsing if they could locate the hyperlink in either the correct section or incorrect section.
This led to frustration and cessation of the tasks due to user fatigue.

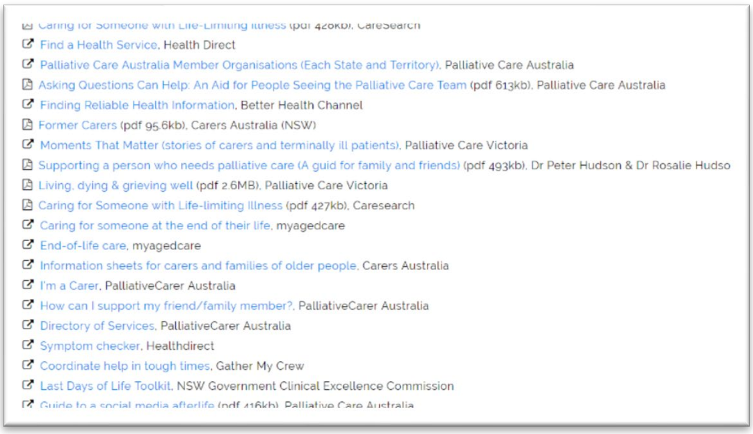

→ This section could be considered a repository of information for all aspects of caring at the end of life but the current organisation structure has been observed to be confusing and the sheer amount of resources overwhelming. Recommend re-ordering resources both between sections and within sections to enhance the user experience in finding information.
Suggest alphabetising the sections or resources within the sections or alternative order them in another manner but provide some structure.

21. MEDIUM: SEARCH FUNCTION WOULD BE HELPFUL TO FIND INFORMATION IN THE CARER LIBRARY IN A MORE DIRECT AND EFFECTIVE MANNER

There were several attempts at locating a search function within the Library page to assist searching as the section headers were often vague or non-specific (included terms such as ‘Populations’, ‘CarerHelp ‘ and particularly ‘Carer Support’ which provided references to resources that were not matched to the header). Participants also suggested that there should be additional sections which were identified as a weakness of the Toolkit including Palliative Care, Legal and Financial, Assistance with Processes. Observation of search behaviour also found that no participants attempted or used the search function in the horizontal menu bar once in the Carer Library.

→ Recommend re-organisation of the sections and addition of sub-headings within sections should assist users to locate the resources more easily. Could feature the search function in the horizonal menu (internal pages) within the Getting Started page (if developed).

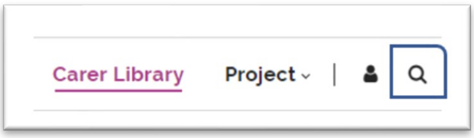

→ Section text labels should reviewed and user-centric language applied to headers as carers will not understand medical jargon or terms commonly directed at health care professionals.

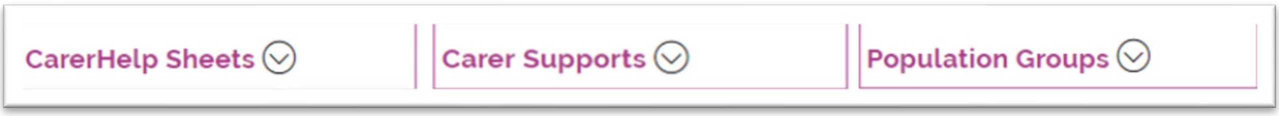

22. LOW-MEDIUM: RESOURCES TO SUPPORT CARERS OF PEOPLE LIVING WITH COMPLEX ILLNESSES TO HELP WITH UNCERTAINTY OF CARING TRAJECTORY

Two of the participants had experienced caring for loved ones with complex illnesses toward the end of their lives and found there was a lack of information for carers in this not uncommon situation. There were suggestions of additional resources that would provide some support and comfort to carers in this situation as there is a lack of information in the wider palliative care sector particularly around uncertainty and the impacts on carers.

Participants suggested additional resources that could assist carers who are looking after complex and difficult conditions that do not follow the identified pathway, particularly as this is a real issue and very distressful as they don't have enough information and reassurance from healthcare professionals of what is going to happen and when. Carers experienced distressing incidents relating to repeated ambulance and hospital admissions and perhaps a medical or condition template that carers could complete which could be handed to the attending HCP which would reduce the repeated "story-telling" which is distressing and upsetting for the carer.

→ A resource would assist these under-serviced group of carers that could be added to the 'Carer Library' or 'Being Prepared' section of the Toolkit.

3.8 LANGUAGE and VISUAL REPRESENTATION

23. HIGH: INFORMATION DISPLAYED IN THE TOOLKIT WAS HARD TO READ AND LIGHT IN CONTRAST TO THE BACKGROUND

Four of the six participants found the text and hyperlinks to be difficult to read due to the font size and the hyperlinks were too light in contrast to the background. This observation could be attributed to the demographics of the usability group with age and visual decline prevalent in this group of carers and could be extrapolated to the larger carer population. None of the four augmented the screen for improved readability.

→ Recommend increasing the text size to minimum 12-13pt and change the font colour of the hyperlinks to a darker blue.

24. HIGH: BALANCE IN LANGUAGE AND TONE WILL BE IMPORTANT FOR THIS GROUP OF USERS PARTICULARLY AS THERE WILL BE VARIATION IN SELF-IDENTIFICATION OF WHERE CARERS ARE IN THE CARING TRAJECTORY, LEVELS OF PREPAREDNESS-ACCEPTANCE-LIVED EXPERIENCE AND TYPES OF INFORMATION REQUIRED BASED ON THE LEVEL OF SUPPORT BEING RECEIVED IN THE COMMUNITY

Through think-aloud methods, observations of carers interacting with the Toolkit has provided a useful insight into the delicate balance between “being gentle” or “fluffy” and being “hard” or “practical” or “telling it like it is” depending on individual carers. Overwhelmingly, carers suggested that there was no point to being gentle or overly delicate because of the lived experience and all the hard stuff that goes with caring, the death and the grieving after death. With this view, there may need to be a consideration to the likely real-life situations that some of the carers will be experiencing that may prompt or are happening to give context to their information seeking and tailor the language to meet their needs.

→ Choice of tone and language should be applied consistently across the Toolkit and into the resources developed by the Project Team.

4. APPENDIX: TECHNICAL REPORT

4.1 USABILITY SESSION SUMMARY

A combination of observation and Think Aloud protocol was undertaken to gather both visual and verbal feedback on the interaction of the participants with the Toolkit as they completed the eight tasks.

Participants were given 5 minutes to complete the task. The activity was stopped when the target information was located, the participant gave up or 5 minutes had passed.

Participants were also asked to provide feedback on other features, information or problems they may have encountered during the session

All issues, problems and feedback were analysed for levels of severity as guided by Nielsen's Severity rating tool (level of severity [number of participants experiencing the problem, level of frustration recovery, frequency of the issues and the influence of the problem on critical tasks]and the time and resources required to modify the problem in the interface).

4.2 TASK SCENARIOS, TASK CHARACTERISTICS, TASK SUMMARY & PARTICIPANT LIST

4.2.1 TASK SCENARIOS

Tasks were developed from the six key objectives identified by the developers of the Carers Toolkit:

1. **An understanding of what to expect when someone is dying**
2. **Access to high quality information and resources that support them in the carer role**
3. **Better communication with the health care team, family and friends**
4. **A greater sense of control over their role**
5. **Greater wellbeing**
6. **Knowledge of the services available to carers**

Each objective was explored by a task with a specified goal being an action or targeted information within the Carers Toolkit.

Test participants were given eight tasks in this study, one at a time:

1. You need some information on how to manage restlessness and confusion in the person you are caring for
2. Locate an indicator that helps *you* to know that this toolkit resource is trustworthy and provides quality information
3. 3A) You need to build a list of the ever-growing issues and problems that you need to discuss with the GP, palliative care team and specialists to take with you when you visit them or to have it handy when they are visiting your home
4. 4A) You have some self-doubt about whether you are able to carer for your loved one, find some information that will help you to make this decision and to aid with discussions
5. You have always been an organised person and finding your way after caring feels messy and full of insecurities.
Find some information that could help you to build a map to assist you to plan for the future.
6. Find information on how to locate palliative care services in your local area
7. 4B) Find some information that will help you to legally make decisions for the person you are caring for
8. 3B) Your fridge and cupboard are covered in paper with various names and numbers to call in case of emergencies or of something unexpected happens.
Find something to organise this information.

4.2.2 PARTICIPANTS IN THE STUDY

Six people participated in the study. All participants have experienced caring for loved ones with a life-limiting condition who were living at home.

Carer	Gender	Age	**Frequency of Web Usage(1)**	**Internet Use(2)**	**Self-Assessment of Technical Ability(3)**	**Device option(4)**	**Health Literacy Level (NVS)ˇ**	**Overall Satisfaction with Prototype (CSUQ)#**<br>**OS:** Overall Satisfaction<br>**USE:** Usefulness<br>**INFOQUAL:** Information Quality<br>**INTERQUAL:** Interface Quality
1	F	36	Daily	• View News or Current Affairs<br>• View Entertainment / Streaming programs (Netflix etc) or You Tube<br>• Social Media or Networking<br>• To pay bills or complete banking transactions<br>• Looking for information from websites or Apps	An Expert who is confident in finding and using online information	• Laptop Computer<br>• Mobile Phone	6	**OS: 3.43**<br>**USE:** 3.33<br>**INFOQUAL:** 3.75<br>**INTERQUAL:** 3.25
2	F	73	Daily	• To pay bills or complete banking transactions<br>• Looking for information from websites or Apps	Having Intermediate skills who is mostly confident	• Laptop Computer<br>• Mobile Phone	6	**OS: 5.86**<br>**USE:** 6.20<br>**INFOQUAL:** 5.00<br>**INTERQUAL:** 6.50
3	F	71	Daily	• View News or Current Affairs<br>• View Entertainment / Streaming programs (Netflix etc) or You Tube<br>• Social Media or Networking<br>• To pay bills or complete banking transactions<br>• Looking for information from websites or Apps	Having Intermediate skills who is mostly confident	Mobile Phone	6	**OS: 4.73**<br>**USE:** 4.83<br>**INFOQUAL:** 4.60<br>**INTERQUAL:** 4.75
4	F	66	Weekly	• View News or Current Affairs<br>• Social Media or Networking<br>• To pay bills or complete banking transactions<br>• Looking for information from websites or Apps	An avoider of everything that Intermediate skills who is mostly confident	Desktop Computer	5	**OS: 2.93**<br>**USE:** 3.00<br>**INFOQUAL:** 3.60<br>**INTERQUAL:** 2.00
5	F	67	Daily	• View Entertainment / Streaming programs (Netflix etc) or You Tube<br>• Social Media or Networking<br>• To pay bills or complete banking transactions	Having Intermediate skills who is mostly confident	Mobile Phone	6	**OS:** 3.87<br>**USE:** 4.00<br>**INFOQUAL:** 4.20<br>**INTERQUAL:** 3.25

				• Looking for information from websites or Apps				
6	F	63	Daily	• View News or Current Affairs<br>• View Entertainment / Streaming programs (Netflix etc) or You Tube<br>• Social Media or Networking<br>• To pay bills or complete banking transactions<br>• Looking for information from websites or Apps	Having Intermediate skills who is mostly confident	• Laptop Computer<br>• Tablet (for example: iPAD)<br>• Mobile Phone	6	**OS:** 2.87<br>**USE:** 3.00<br>**INFOQUAL:** 2.80<br>**INTERQUAL:** 2.75

Frequency of Web Usage[(1)]: a) Daily, b) Once or Twice a Week, c) Weekly, d)Once or Twice a Month, e) Rarely or hardly ever
Internet Use[(2)]: a) View News or Current Affairs, b) View Entertainment / Streaming programs (Netflix etc) or You Tube, c) Social Media or Networking (Facebook etc), d) Pay bills or complete banking transactions, e) Looking for information from websites or Apps
Self-Assessment of Technical Ability[(3)]: a) Avoider of everything that is online – you would prefer to find a 'real' person to help, b) Novice or Learner or Beginner, c) Having Intermediate skills who is mostly confident, d) Expert who is confident in finding and using online information
Device option[(4)]: a) Smart TV, b) Desktop Computer, c) Laptop Computer, d) Tablet (for example: iPAD), e) Mobile Phone
NVS (Newest Vital Sign)ˇ: interpreted as scores of [0-1] high likelihood of limited health literacy, [2-3] possibility of limited literacy and [4-6] almost always indicates adequate literacy
Overall Satisfaction of Use (CSUQ)[#]: Average scores from 15 item scale (measured 7-point Likert scale 1 = Strongly Agree to 7 = Strongly Disagree)

4.2.3 TASK CHARACTERISTICS

Participants were given 2 minutes to browse the Carer Toolkit prior to the tasks being undertaken.

**Objective - Task Number**	**Completion Rate*** % Success (Success:Failure)	**How Easy was it to complete the task** Self- Assessed (average score n=6) Very Difficult (1) – Very Easy (7)	**How confident are you that you completed the activity fully?** Self- Assessed (average score n=6) Low (1) – High (7)	**What was your level of satisfaction after completing this task?** Self- Assessed (average score n=6) Low (1) – High (7)
1 – 1	67.7% (4:2)	4.33	5.00	4.83
2 – 2	100.0% (6:0)	6.00	6.17	6.50
3A – 3	50.0% (3:3)	4.00	3.33	3.50
4A – 4	67.7% (4:2)	5.83	5.83	6.0
5 – 5	33.3% (2:4)	4.67	5.33	5.33
6 – 6	33.3% (2:4)	3.00	4.67	4.67
4B – 7	0% (0:6)	4.00	6.00	5.67
3B – 8	100.0% (6:0)	6.33	6.67	6.67

*Participants were given maximum of 5 minutes to complete the task. Task was considered a success if the user was able to locate the information within the time period. Task failure was if the user was unable to find the information in the allotted time, if the task was stopped due to the user giving up or if the user nominated that they would have stopped looking and left the Toolkit to search for this information via a different mechanism (such as performing a 'Google Search').

4.2.4 TASK SUMMARY

Objective - Task Number	Target / Specified Action or Information	Comments	Completion Rate* % Success (Success:Failure)
1 – 1	Locate information about symptoms and management of these toward the end of life (information located in the 'Symptoms' section of the Carer Library (https://www.carerhelp.com.au/tabid/5615/Default.aspx#PS) or Confusion and Terminal Restlessness PDF found on the 'Caring for the Dying' webpage (https://www.carerhelp.com.au/tabid/5614/Default.aspx)	• All but two carers were able to locate some information on managing the symptoms of confusion (delirium) and restlessness in the dying person.<br>• 1 of the 5 carers found the information in the 'Caring for the Dying' section of the website, all others found this information in the Carer Library under 'Symptoms'<br>• 1 carer found some information in Managing Symptoms LM, 1 searched the CL but then would have moved to do a 'Google Search' as could not find any reference to confusion or restlessness.<br>• No participants referred to the Symptom Management videos in Caring for the Dying section.<br>• Users searching within the CL for this information were frustrated commenting that there was too much information, which was muddled within sections and the page was very overwhelming.	67.7% (4:2)
2 – 2	Find indicators of trust and quality, could include institution logos, privacy or funding statements, 'About the Project' webpage (https://www.carerhelp.com.au/tabid/5675/Default.aspx)	• All six participants could locate an indicator of quality and trust<br>• 1 participant located the institutional logos on the Home Page<br>• 4 users located the 'About the Project' page and noted the funding and organisations involved<br>• 2 carers referred to the Privacy Statement and one checked the e-mail address for consistency	100.0% (6:0)
3A – 3	Locate and download 'Things to discuss with your healthcare team' document on the 'Being Prepared' webpage (https://www.carerhelp.com.au/tabid/5613/Default.aspx)	• Only 3 of the 6 carers could locate this item in the TK<br>• 1 carer was able to navigate directly to this PDF using the horizontal menu, the other two used the Carer Pathway page but only after trying to find the document in the CL or CV page<br>• Indicates issues with the HP, search functions and general menu structures due to the navigation problems of the users	50.0% (3:3)
4A – 4	Find and download the My Plan for Moving Forward PDF found within the 'After Caring' webpage (https://www.carerhelp.com.au/tabid/5577/Default.aspx)	• 4 carers were able to find this PDF in the 'After Caring' webpage<br>• 1 carer used the 'Hamburger Menu' on the HP, the other 3 used either the 'Carer Pathway' or 'Carer Library' buttons on the HP to enter the site as there were no other viable options – they then used the horizontal menu within the site to navigate to the page<br>• 1 carer found the after caring video on the Carer Voice page and though that there might be some information within this resource that may help bereaved carers	67.7% (4:2)

5 – 5	Complete the Carer Preparedness Scale found on 'Being an End of Life Carer' webpage at https://www.carerhelp.com.au/tabid/5742/Default.aspx	• 2 of the 6 carers were able to find this tool within the 'Being an EoL Carer' page<br>• 2 carers gave up looking for this tool after not being able to find it in the sections that they thought it may have been<br>• 2 carers located the 'Tips for Carers' PDF in the key resources section but bypassed the link to the tool on the same page<br>• All six carers had the opportunity to use the tool, all thought this was a useful tool for some carers and should be prominent in the Being an EoL Carer and Being Prepared pages as the caring journey does change with increasing complexities, good tool to re-assess you capabilities.<br>• All thought the premise of the tool and it's output was a great idea and liked that you could print or e-mail the results.	33.3% (2:4)
6 – 6	Find the Palliative Care Australia Services Directory Link in the CL under General Resources and 'Tools and Resources' sections https://www.carerhelp.com.au/tabid/5615/Default.aspx#GR	• 2 of the participants were able to locate the link in the CL<br>• All participants were perplexed to why there was no mention of palliative care or accessing palliative care in the TK<br>• All commented on the importance of palliative care in the role of supporting care particularly for those with limited knowledge of services available, when and how to engage with this support<br>• All participants would like to see references to palliative care services and their role in the support of the carer (from this aspect not so much for the person dying) especially with reference to networking with organisations or service providers	33.3% (2:4)
4B – 7	Locate some information on the legal requirements of making decisions for someone else (found at 'Making Healthcare Decisions for Someone Else' on https://www.carerhelp.com.au/tabid/5615/Default.aspx#GR	• None of the participants located this information during the test although all 6 carers directed their search to the 'Sorting out financial matters' section in the 'Being Prepared' (https://www.carerhelp.com.au/tabid/5613/Default.aspx) webpage<br>• All 6 carers emphasized the importance of this information and stressed legal information should not be hidden in the CL rather should sit alongside the financial matters in the key resources section.	0% (0:6)
3B – 8	Find the 'Emergency Checklist' found on 'Being Prepared' webpage or in CL (https://www.carerhelp.com.au/tabid/5613/Default.aspx) or the 'Who to Call List' on the 'Caring for the Dying' webpage (https://www.carerhelp.com.au/tabid/5614/Default.aspx)	• All 6 carers were able to find the 'Emergency Checklist' PDF and were able to open this document<br>• None of the carers located the 'Who to Call List' in the Caring for the Dying section.<br>• 4 carers found this document in the 'Being Prepared' page using the horizontal menu on the inside pages after using the buttons on the HP<br>• 2 carers found this document in the CL in 'CarerHelp' resource section, using the 'Carer Library ' button on the HP<br>• All participants thought this was a useful and relevant resource that all carers could use and liked that you could print this out to use	100.0% (6:0)

HP = Home Page, CL = Carer Library, LM = Learning Module, CV = Carer Voice, TK = Carers Toolkit
